# Early infantile epileptic encephalopathy due to biallelic pathogenic variants in *PIGQ*: Report of seven new subjects and review of the literature

**DOI:** 10.1002/jimd.12278

**Published:** 2020-08-03

**Authors:** Devon L. Johnstone, Thi Tuyet Mai Nguyen, Jessica Zambonin, Kristin D. Kernohan, Anik St‐Denis, Nissan V. Baratang, Taila Hartley, Michael T. Geraghty, Julie Richer, Jacek Majewski, Eric Bareke, Andrea Guerin, Manuela Pendziwiat, Loren D. M. Pena, Hilde M. H. Braakman, Karen W. Gripp, Andrew C. Edmondson, Miao He, Rebecca C. Spillmann, Erik A. Eklund, Allan Bayat, Hugh J. McMillan, Kym M. Boycott, Philippe M. Campeau

**Affiliations:** ^1^ Children's Hospital of Eastern Ontario Research Institute Ottawa Ontario Canada; ^2^ Research Center, CHU Sainte Justine University of Montreal Montreal Quebec Canada; ^3^ Department of Genetics Children's Hospital of Eastern Ontario Ottawa Ontario Canada; ^4^ Division of Metabolics and Newborn Screening, Department of Pediatrics Children's Hospital of Eastern Ontario Ottawa Ontario Canada; ^5^ Department of Human Genetics McGill University Montreal Quebec Canada; ^6^ McGill University and Genome Quebec Innovation Centre Montreal Quebec Canada; ^7^ Division of Medical Genetics, Department of Pediatrics Queen's University Kingston Ontario Canada; ^8^ Department of Neuropediatrics Christian‐Albrechts‐University of Kiel Kiel Germany; ^9^ Division of Human Genetics Cincinnati Children's Hospital Medical Center Cincinnati Ohio USA; ^10^ Department of Pediatrics University of Cincinnati College of Medicine Cincinnati Ohio USA; ^11^ Department of Neurology Academic Center for Epileptology Kempenhaeghe & Maastricht University Medical Center Heeze The Netherlands; ^12^ Department of Pediatric Neurology, Amalia Children's Hospital Radboud University Medical Center & Donders Institute for Brain, Cognition and Behaviour, Radboud University Nijmegen The Netherlands; ^13^ Division of Medical Genetics A. I. DuPont Hospital for Children/Nemours Wilmington Delaware USA; ^14^ Department of Pediatrics, Division of Human Genetics Children's Hospital of Philadelphia Philadelphia Pennsylvania USA; ^15^ Department of Pathology and Laboratory Medicine Children's Hospital of Philadelphia Philadelphia Pennsylvania USA; ^16^ Division of Medical Genetics, Department of Pediatrics Duke University Medical Center Durham North Carolina USA; ^17^ Department of Pediatric Neurology, Region Skåne and Clinical Sciences Lund University Skåne University Hospital (SUS) Lund Sweden; ^18^ Department of Genetics and Personalized Medicine Danish Epilepsy Centre Dianalund Denmark; ^19^ Institute for Regional Health Services Research University of Southern Denmark Odense Denmark; ^20^ Division of Neurology, Department of Pediatrics Children's Hospital of Eastern Ontario Ottawa Ontario Canada; ^21^ Department of Pediatrics, Sainte‐Justine Hospital University of Montreal Montreal Quebec Canada

**Keywords:** epileptic encephalopathy, exome sequencing, GPI, IGD, *PIGQ*, rare diseases

## Abstract

We investigated seven children from six families to expand the phenotypic spectrum associated with an early infantile epileptic encephalopathy caused by biallelic pathogenic variants in the phosphatidylinositol glycan anchor biosynthesis class Q (*PIGQ*) gene. The affected children were all identified by clinical or research exome sequencing. Clinical data, including EEGs and MRIs, was comprehensively reviewed and flow cytometry and transfection experiments were performed to investigate PIGQ function. Pathogenic biallelic *PIGQ* variants were associated with increased mortality. Epileptic seizures, axial hypotonia, developmental delay and multiple congenital anomalies were consistently observed. Seizure onset occurred between 2.5 months and 7 months of age and varied from treatable seizures to recurrent episodes of status epilepticus. Gastrointestinal issues were common and severe, two affected individuals had midgut volvulus requiring surgical correction. Cardiac anomalies including arrythmias were observed. Flow cytometry using granulocytes and fibroblasts from affected individuals showed reduced expression of glycosylphosphatidylinositol (GPI)‐anchored proteins. Transfection of wildtype *PIGQ* cDNA into patient fibroblasts rescued this phenotype. We expand the phenotypic spectrum of *PIGQ*‐related disease and provide the first functional evidence in human cells of defective GPI‐anchoring due to pathogenic variants in *PIGQ.*

**Abbreviations**
ALP: alkaline phosphatase
CHO: Chinese hamster ovary
DWI: diffusion weighted imaging
EEG: electroencephalogram
EIMFS: epilepsy of infancy with migrating focal seizures
FLAER: fluorescein‐labeled proaerolysin
GDD: global developmental delay
GPI: glycosylphosphatidylinositol
GPI‐AP: GPI anchored protein
IGD: inherited GPI disorder
MRI: magnetic resonance imagining
WT: wildtype

**SYNOPSIS**

We expand the phenotypic spectrum of *PIGQ*‐related disease and provide the first functional evidence in human cells of defective GPI‐anchoring due to pathogenic variants in *PIGQ*.

## 1 INTRODUCTION

The GPI‐anchor anchors more than 150 proteins to the cell surface.^1^ These GPI‐anchored proteins (GPI‐APs) play important roles in embryogenesis, cell signaling, immune response and neurogenesis.^2^, ^3^, ^4^, ^5^ Thirty‐one different genes are involved in the biosynthesis of the GPI‐anchor,^6^ of which 22 genes have been associated with human disease.^7^ Clinical features generally include global developmental delay (GDD), epileptic seizures, hypotonia, and congenital anomalies.^2^ The clinical spectrum is likely related to the various cellular functions of GPI‐APs, which include adhesion molecules, complement regulatory proteins, hydrolytic enzymes, protease inhibitors, and receptors.^1^, ^8^

Phosphatidylinositol glycan anchor biosynthesis class Q (PIGQ) is a key co‐enzyme in the N‐acetylglucosamine transferase complex, the first step catalyzing the biosynthesis of the GPI‐anchor,^9^, ^10^, ^11^ and is encoded by *PIGQ* (OMIM 605754).^12^ To date, three patients with *PIGQ* variants have been described,^13^, ^14^, ^15^ however, for the first two patients, limited clinical information was published. Martin et al showed that *PIGQ‐*deficient Chinese hamster ovary (CHO) cells transfected with human *PIGQ* cDNA lacking exon 3 did not restore surface expression of GPI‐APs as well as wildtype (WT),^13^ however, functional data in human cells remained elusive. Recently, Starr et al^15^ provided a detailed clinical description of a patient with compound heterozygous variants in *PIGQ*. Very little is known about the clinical and functional spectrum associated with *PIGQ* insufficiency.

Herein, we describe and expand the phenotypical and neurophysiological spectrum of PIGQ deficiency in seven affected individuals and show reduced expression of GPI‐APs in patient granulocytes and fibroblast cell lines, with reversibility using *PIGQ* cDNA.

## 2 METHODS

### 2.1 Patient recruitment

Ethics approvals were obtained from the local institutional review boards and informed consent was obtained from patients' legal guardians. Details of local ethics approvals are available in Supporting Information. Collaboration was made possible using Matchmaker Exchange.^16^ Detailed clinical examinations, magnetic resonance imaging (MRIs) and electroencephalograms (EEGs) were collected for each subject. Here forth, we abbreviate each subject to “St” (St1, St2, and so on).

### 2.2 Exome sequencing

DNA was extracted from whole blood, purified, and analyzed using an exome sequencing trio approach when both parents were available. All exomes were aligned to the human reference genome GRCh37/hg19. Detailed protocols for exome capture, sequencing, pipeline and analysis for each patient/family are available in Supporting Information. Primers for Sanger sequencing confirmation of variants are found in Table S1.

### 2.3 Cell line establishment

For St3b, a skin biopsy was taken and sent to the Centre for Applied Genomics (Toronto, Canada), for establishment of a fibroblast cell line. A skin biopsy for St5 was similarly used for establishment of a fibroblast cell line at the Duke Cytogenetics Laboratory. A fibroblast cell line for St2 was established independently at the Children's Hospital of Philadelphia.

HyClone DMEM media (GE Healthcare Life Sciences) supplemented with 10% fetal bovine serum, penicillin‐streptomycin (SV30010, GE Healthcare Life Sciences) and 2 mM l‐glutamine (SH30033401, Thermo Scientific), was used for cell‐line maintenance.

### 2.4 Flow cytometry

Fresh blood samples from St4 and St5, as well as from healthy controls were stained with the GPI‐AP markers: PE‐conjugated anti human CD16 (BioLegend), FITC‐conjugated mouse anti human CD55 and CD59 (BD Pharmingen), or Fluorescein‐labeled proaerolysin (FLAER)‐Alexa 448 (Cedarlane) for 1 hour on ice. Red blood cells were lysed in FACS Lysing Solution (BD Bioscience). Granulocytes from St1 as well as a control were stained with FLAER and analyzed by flow cytometry in a clinical lab.

For fibroblasts, cells were harvested at 80% to 90% confluency, stained with FLAER‐Alexa 448, FITC‐conjugated mouse anti human CD73 (BioLegend) or PE‐ conjugated mouse anti human CD109 (BioLegend) for 1 hour on ice in the incubation buffer containing 0.5% BSA, then fixed in 3.7% formaldehyde. For all assays, non‐specific binding was washed off before analyzing by a BD FACSCanto II system (BD Biosciences) followed by the FlowJo software analysis. We use at least three different controls at two different times for fibroblasts (before and after the rescue). Fibroblast characteristics may change with time in culture and with passages, we thus use for our analysis and figures the control sample which displayed similar cell population patterns (eg, size and granularity) as the test's fibroblasts. Fibroblasts established from St2 and a control were stained with FITC‐CD59 and analyzed by flow cytometry in an independent lab (see Supporting Information).

### 2.5 Rescue assays of GPI‐APs on fibroblasts

Lentiviruses carrying a wildtype *PIGQ*‐Lv105 (NM_004204.3) or an empty‐Lv105 construct (GeneCopoeia) with the presence of packaging plasmids pMD2.G and psPAX2 (AddGene) were produced in HEK293T cells. Fibroblasts (established from St3b and St5) were transduced with the lentiviruses (typically with transduction efficiency over 25%), and selected by Puromycin resistance, creating a stable cell line. Untransduced cells, rescued cells and control cells were subjected to flow cytometry analyses as described above for fibroblasts.

## 3 RESULTS

We identified seven affected individuals with biallelic *PIGQ* variants from six unrelated families. The cohort comprised of five females and two males, from various ancestries. Detailed clinical summaries can be found in Supporting Information as well as Table 1 and Table S2. EEG descriptions are found in Table S3, and MRI findings are summarized in Table S4.

**TABLE 1:** Clinical features of seven new affected individuals from six families with biallelic variants in *PIGQ*, and review of the literature

Subject ID/source	St1	St2	St3a	St3b	St4	St5	St6
Variants & Inheritance (NM_148920.2)	Homozygous: c.1611del p.R538Afs*24	Maternal: c.1199_1201del p.Y400del Paternal: c.942+1G>A IVS4+1G>A	Maternal: c.1578_1579del p.Q527Afs*75 Paternal: c.1199_1201del p.Y400del	Maternal: c.1578_1579del p.Q527Afs*75 Paternal: c.1199_1201del p.Y400del	Maternal: c.1130_1168del p.A377_S389del Paternal: c.1345G>C p.G449R	Maternal: c.49G>A p.G17R Paternal: c.942+1G>A IVS4+1G>A	Homozygous: c.1670del p.G557Dfs*4
Gender	Female	Female	Female	Female	Female	Male	Male
Ancestry	Turkish	European/Puerto Rican	British Isles/French Canadian	British Isles/French Canadian	Lebanese/Iraqi	Mexican	Afghani
Current age	11 y	6 y 6 mo	Deceased 2 d	Deceased 5 y	Deceased 9 m	2 y 2 m	Deceased 3 y 9 m
Prenatal issues	−	Polyhydramnios	Prominent kidneys, premature rupture of membranes	−	Polyhydramnios, hepatomegaly, hydronephrosis	Dandy Walker malformation	−
Neonatal complications	−	Respiratory distress, hypoglycemia, failed newborn hearing screen in left ear.	Respiratory distress, renal and cardiac failure	Jaundice, secundum atrial ventricular defect detected after birth	Respiratory distress	Respiratory distress, feeding difficulties, jaundice, PDA, PFO	Feeding difficulties, hypertonia
Developmental delay	+	+	N/A	+	+	+	+
Seizure onset	6 mo	7 mo	N/A	Almost 4 mo	7 mo	6 mo	2.5 mo
Hypotonia	+	+	N/A	+	+	+	+
Abnormal movements	+	+	N/A	+	+	+	+
Facial dysmorphism	−	+	+	+	+	+	+
Cranial shape anomalies	−	+	−	−	+	+	+
Teeth anomalies	−	+	N/A	+	Too young	+	−
Skeletal anomalies	+	+	−	+	+	+	−
Joint contractures	+	−	−	+	+	−	−
Other dysmorphic features	+	−	+	−	+	−	−
Deafness	−	−	−	−	Mild left conductive	−	−
Ophthalmological anomalies	+	+	N/A	+	+	+	+.
Cardiac anomalies	−	+	+	+	+	+	+.
Genitourinary abnormalities	+	+	+	No U/S done	+	+	+
Gastrointestinal issues	+	+	N/A	+	+	+	+
Serum alkaline phosphatase	Normal	Intermittently elevated	Not measured	Elevated	Elevated	Not measured	Normal

Subject ID/source	Martin et al^13^	Alazami et al^14^	Starr et al^15^
Variants & Inheritance (NM_148920.2)	Homozygous: c.690‐2A>G	Homozygous: c.619C>T p.R207*	Maternal: c.968_969del p.L323Pfs*119 Paternal: c.1199_1201del p.Y400del
Gender	Male	N/A	Male
Ancestry	West African	N/A	N/A
Current age	Deceased 2 y 4 m	N/A	Deceased 10 mo
Prenatal issues	−	N/A	Severe polyhydramnios requiring multiple amniocentesis fluid reduction procedures
Neonatal complications	At 4 wk: cyanotic episodes with eye twitching, brief stiffening of upper body.	N/A	Feeding difficulties, trembling episodes
DD/ID	+	+	+
Seizure onset	4 wk	N/A	7 mo
Hypotonia	+	N/A	+
Abnormal movements	+	N/A	+
Facial dysmorphism	+	N/A	+
Cranial shape anomalies	−	N/A	+
Teeth anomalies	−	N/A	−
Skeletal anomalies	−	N/A	+
Joint contractures	−	N/A	N/A
Other dysmorphic features	+	N/A	+
Deafness	−	N/A	−
Ophthalmological anomalies	+	+	+
Cardiac anomalies	−	N/A	+
Genitourinary anomalies	−	N/A	+
Gastrointestinal issues	+	N/A	+
Serum alkaline phosphatase	N/A	N/A	Elevated

*Note:* Further details are provided in Table S2.

Abbreviations: N/A, not applicable; PDA, patent ductus arteriosus; PFO, patent foramen ovale; U/S, ultrasound.

### 3.1 Variants identified

**FIGURE 1:**
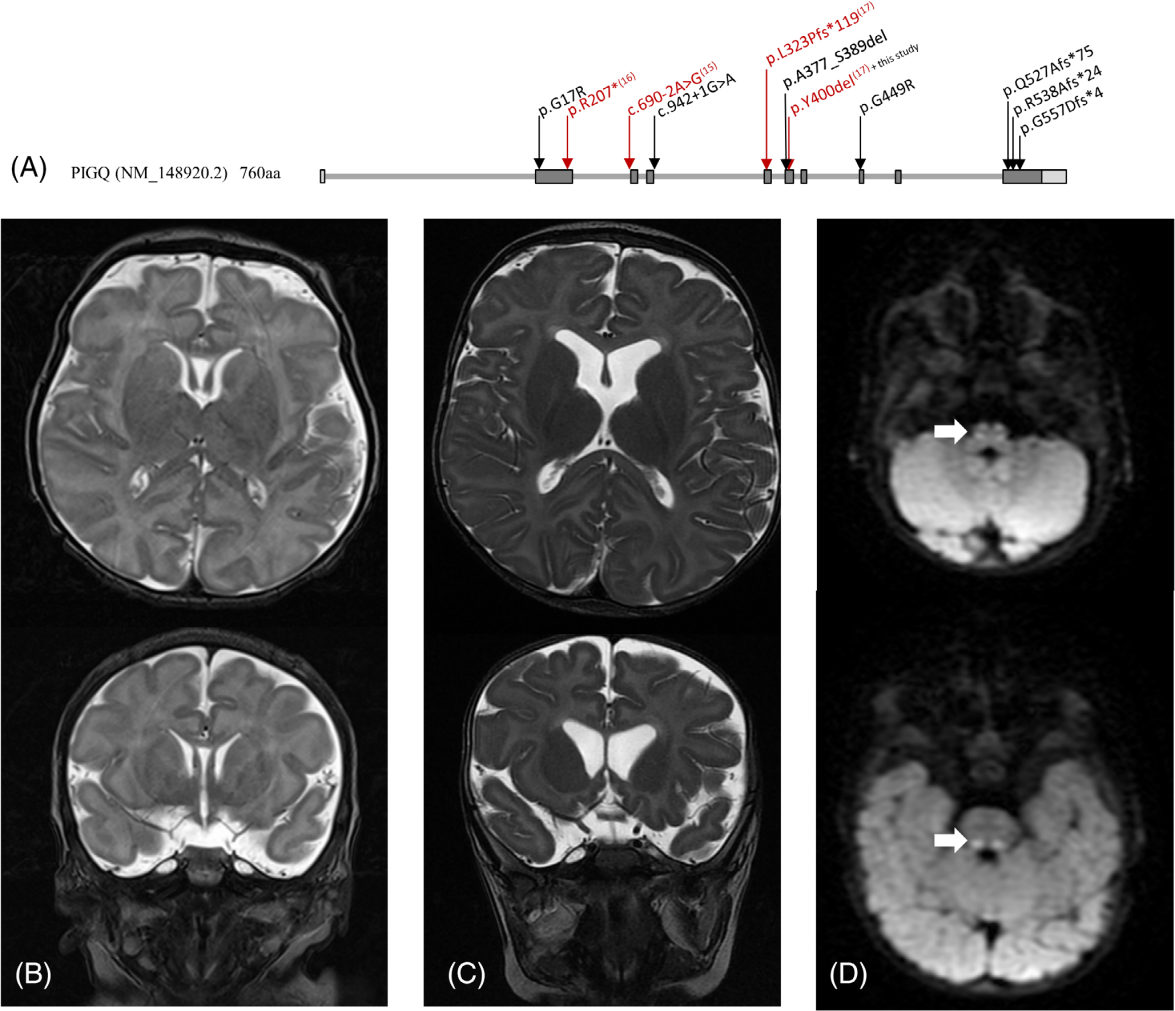
A, Variants in *PIGQ* identified in this study localized on isoform 1 (NM_148920.2) of the gene. Variants reported in previous studies are in red with corresponding reference numbers in superscript. Brain MRI of subject 4 was performed at age 9 days, B, and 7 months, C. At each age, T2‐weighted image sequences were performed with axial (top) and coronal (bottom) images shown. Brain MRI at 7 months showed progressive cortical volume loss with increased prominence of the lateral ventricles, consistent with loss of subcortical white matter volume compared to the prior study. Mild delay in myelination was apparent. D, Diffusion weighted axial images identified restricted diffusion in the medial lemniscus tracts, bilaterally

We identified seven previously unreported *PIGQ* (NM_148920.2) variants predicted to be damaging and one previously identified variant (p.Y400del).^15^ All were either in a homozygous or compound heterozygous state. The novel variants included two missense variants (p.G17R; p.G449R), a canonical splice site substitution (c.942+1G>A), an in‐frame deletion (p.A377_S389del) and three frameshifts (p.Q527Afs*75, p.R538Afs*24 and p.G557Dfs*4) (Figure 1A). Variants identified in other genes are listed in the detailed clinical descriptions in Supporting Information but were excluded as disease‐causing due to phenotype incompatibility, predicted benign pathogenicity, or mode of inheritance.

### 3.2 Phenotypic analysis

There was significant morbidity and mortality associated with biallelic *PIGQ* variants. Four of the seven patients died prematurely (St3a at 2 days of age secondary to cardiac and renal failure, St3b at 5 years of age due to asystole, St4 at 9 months of age under palliation, and St6 at 3 years and 9 months of age due to recurrent pneumonias). A presumed affected older brother of St5 died a few hours after birth, but DNA was not collected so the relationship to the familial variants cannot be confirmed.

Abnormal prenatal findings included polyhydramnios (St2 and St4), prominent kidneys and premature rupture of membranes at 28 weeks (St3a), severe fetal hepatomegaly and hydronephrosis (St4) and enlarged lateral ventricles and hypoplastic cerebellar vermis suggestive of a Dandy Walker anomaly (St5). Neonatal complications were present in six patients, including respiratory distress (St2, St3a, St4, and St5), jaundice (St3b and St5), heart failure (St3a), atrial ventricular defect (St3b), patent ductus arteriosus and patent foramen ovale (St5), hypoglycemia (St2) and feeding difficulties with irritability and limb hypertonia of the extremities (St2).

Age at onset of epileptic seizures in the remaining six patients ranged from 2.5 to 7 months of age. Seizure types included focal tonic seizures (St1, St2, and St6, evolving to focal status epilepticus for St2 and St6), bilateral tonic‐clonic (St2, St4, St5, and St6) evolving to status epilepticus (St4 and St6), myoclonic jerks (St3b and St5), epileptic spasms (St4), absence seizures (St1) and migrating focal seizures (St6) (Table S2). Seizure outcome ranged from partially controlled with occasional breakthroughs with illness (St1 and St2), to recurrent status epilepticus (St4, St6), daily focal seizures (St6) or daily clustered myoclonic jerks (St5). Seizures often clustered (St1, St3b, and St5). Notably, St3b was seizure‐free from 4 months until age 2 years, but thereafter had seizures in cycles that would progressively worsen and taper off, with seizure‐free periods of 7 to 12 days (Table S2).

All six patients had a severe to profound GDD. Axial hypotonia was observed in all patients (Table 1, Table S2). Appendicular hypotonia was observed in St2 and St5, whereas appendicular hypertonia was observed in the remaining patients. Severe head lag was observed in St2, St5, and St6. Stereotypic movements, opisthotonos, hyperkinetic movements, or random non‐epileptic jerks were present in all patients (Table S2).

Visual problems were diagnosed in all patients that survived the neonatal period. St3b, St4, St5, and St6 were diagnosed with cortical visual impairment. St1 had short fixation/tracking abilities, whereas an electroretinogram for St2 suggested impaired photoreceptor transmission in the retina. St4 had a mild unilateral conductive hearing deficit, whereas the other subjects had normal hearing (Table S2).

Six of the seven patients had facial dysmorphisms including coarse facies and macroglossia. Four (St2, St4, St5, and St6) had cranial shape anomalies and three (St1, St2, and St3b) had pectus excavatum. Other findings included inverted nipples (St1), scoliosis (St2), delayed dentition (St2, St3b, and St5), bilateral pulmonary interstitial emphysema (St3a), an accessory spleen (St3a), increased nuchal redundancy, short stature, hyperextension of the interphalangeal joints and deep palmar and plantar creases (St4), and an enlarged pinna (St5) (Table S2).

Cardiac anomalies were observed in six patients, including prolapsed mitral valve (St2), pulmonary stenosis (St5), pulmonary hypertension (St6), heart block and arrythmia (St2, St3b, and St4), and right ventricular hypertrophy with poor heart function (St3a, though hypertrophy was not found on autopsy) (Table S2). Genitourinary issues included bilateral hydronephrosis (St4, St5), renal stones with slightly enlarged kidneys (St4) and vesicoureteral reflux (St5). Autopsy of St3a revealed dilated/tortuous ureters with hypoplastic renal pelvis and calyces. Most patients had significant gastrointestinal issues including G‐tube dependency (St2, St3b, St4, St5, and St6), constipation (St1, St2, and St6), idiopathic volvulus (St4, who also had superior mesenteric artery and vein reversal, and St5 who had a markedly dilated colon; both had Ladd's procedure), and duodenal web (St5) (Table S2).

EEG findings were in keeping with clinical presentations and ranged from normal findings early in the disease course, to burst‐suppression patterns, background slowing with interictal multifocal sharp waves, hypsarrhythmia and ictal focal spike and slow wave complexes (Table S3).

MRI findings showed no initial abnormalities in the cerebral cortex, but subsequent imaging showed progressive cortical volume loss (St4, Figure 1B/C; St5). There was poor or incomplete myelination or demyelination in four of the six subjects with available imaging study results (St1, St3b, St4, and St5; Table S4). While St3a did not have brain imaging studies, an autopsy showed pyknotic nuclei of the cerebral cortex, as well as ischemic white matter changes with necrosis and karyorrhexis. There was a strongly positive glial fibrillary acidic protein signal and no striking anoxic changes, giving an overall impression of periventricular leukomalacia. Additional MRI findings included broad periventricular spaces (St1 and St3b), prominent frontal horns of the lateral ventricles (St2), volume loss of the vermis (St3b and St5), dangling choroids (St5) and pituitary hypoplasia with preservation of the stalk (St5; Table S4). MRS showed borderline lactate peaks (St2) and diffusion‐weighted imaging (DWI) showed increased intensity in the bilateral medial lemniscus tracts (St4, Figure 1D; Table S4).

Serum alkaline phosphatase levels were slightly elevated in St3b, and intermittently slightly elevated in St2 and St4 (Table S2). There were no obvious bone abnormalities associated.

### 3.3 Flow cytometry

**FIGURE 2:**
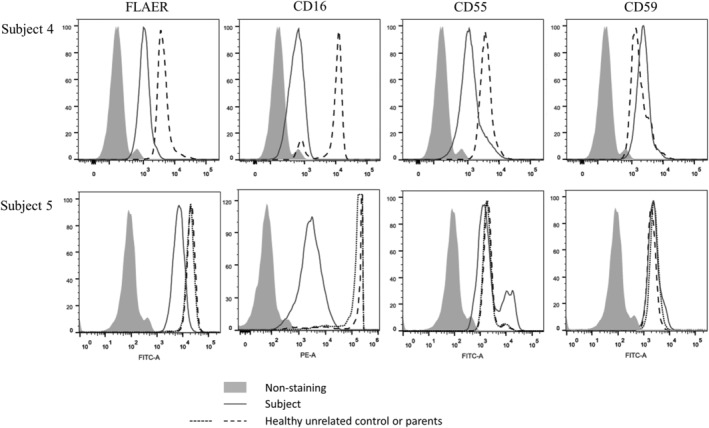
Impact of the *PIGQ* variants on the expression of GPI‐APs. Flow cytometry analysis of granulocytes from fresh blood from subject 4 (top row) and subject 5 (bottom row) and compared to healthy controls and parents, respectively. Fluorescently labeled proaerolysin “FLAER” directly binds the GPI‐anchor, whereas CD16, CD55, and CD59 all stain for GPI‐APs

In blood, both St4 and St5 had very low levels of FLAER and CD16 cell surface localization. In brief, granulocytes from St4 had only 6% the level of FLAER compared to normal while this marker in St5 was 35% (mean fluorescence intensity). For CD16, expression was 28% compared to normal for St4, and 20% in St5. CD55 and CD59 in St5 were normal whereas CD55 was decreased to 68% in St4. However, this patient has an increase in CD59 (Figure 2). Analysis of granulocytes by a clinical lab for St1 showed that the level of FLAER was 64.7% relative to the control.

**FIGURE 3:**
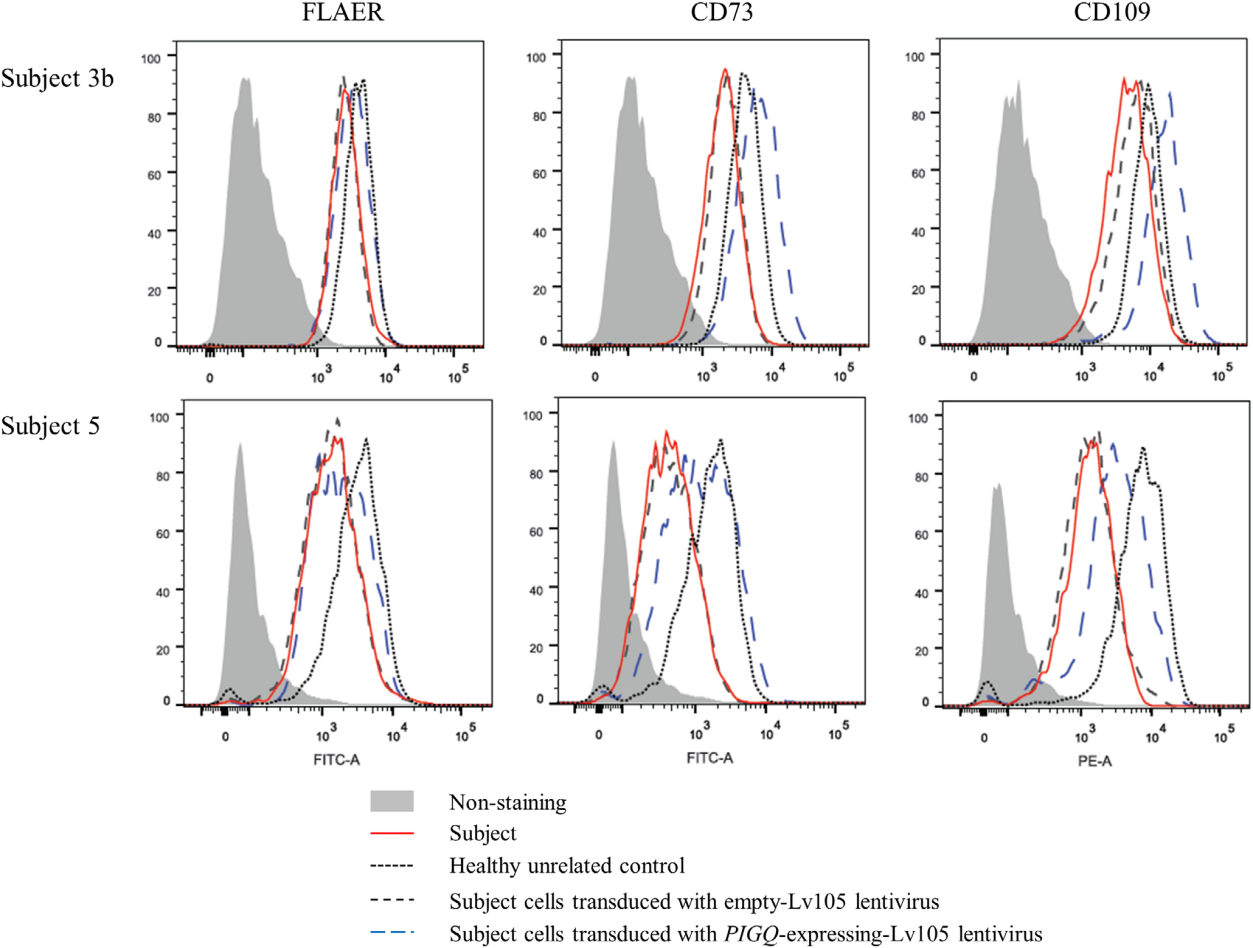
Impact of the *PIGQ* variants on the expression of GPI‐APs. Flow cytometry analysis of fibroblasts derived from subject 3b (top row) and subject 5 (bottom row) and compared to healthy controls and parental derived cell‐lines, respectively. Subject cell lines were further transfected with empty lentivirus or lentivirus expressing WT *PIGQ* cDNA. Fluorescently labeled proaerolysin “FLAER” directly binds the GPI‐anchor, whereas CD73 and CD109 stain for GPI‐APs

For fibroblasts, levels of all markers were decreased in St3b and St5. St3b showed decreases in FLAER, CD73 and CD109 to 45%, 56%, and 20%, respectively relative to control, and these markers in St5 were reduced to 45%, 28%, and 20%, respectively (Figure 3). Analysis of CD59 expression in an independent lab showed a mean fluorescence intensity of 65.2% in St2‐derived fibroblasts relative to the control (Figure S2). While transduction with an empty lentivirus did not affect GPI‐AP levels, a lentivirus expressing wildtype *PIGQ* completely restored GPI‐AP expression in St3b, and there was partial rescue in St5 (FLAER, CD73, and CD109 were increased to 56%, 70%, and 46% vs control; Figure 3).

## 4 DISCUSSION

This cohort of seven new individuals affected by biallelic pathogenic variants in *PIGQ* enables us to broaden the phenotypic spectrum of this rare condition (Tables 1 and S2) and to demonstrate the impact of *PIGQ* on GPI‐anchored proteins.

Patients with biallelic *PIGQ* variants show a broad phenotypic spectrum including epileptic seizures, GDD, hypotonia, feeding difficulties, and multiple congenital anomalies (Tables 1 and S2), clinical features also observed in other inherited GPI‐deficiencies (IGDs).^2^ Most affected individuals with biallelic *PIGQ* variants have delayed myelination, but generally lack gross structural lesions on MRI. Of note, while the affected individual reported by Starr et al^15^ had significant left‐sided ventriculomegaly, we did not observe this in our cohort. A limiting factor could be that our subjects generally underwent MRI before the age of 10 months and that a long‐term follow‐up of MRI findings was not available. Individuals with pathogenic variants affecting other genes in the pathway have shown development of both cerebral and cerebellar loss of tissue over time.^17^, ^18^

While we also did not observe long bone radiolucent lesions as previously described,^15^ skeletal anomalies and delayed dentition were common. Of note, St4 and St5 had midgut volvulus requiring surgery, and St5 had a duodenal web requiring resection. Furthermore, our cohort showed a variety of cardiac issues, including arrhythmia and fatal heart block.

Seizure type and control were highly variable (Tables S2 and S3). Martin et al reported a burst‐suppression pattern on EEG and Ohtahara syndrome,^13^ which was not observed in subsequent patients, but has been reported in other IGDs,^19^ Two patients developed epileptic spasms and St3b showed a recurrent pattern of seizure activity. Interestingly, St6 was diagnosed with epilepsy of infancy with migrating focal seizures (EIMFS), which is a very severe epileptic syndrome with poor outcome, previously known as malignant migrating partial seizures of infancy or Coppola‐Dulac syndrome.^20^ The most common genetic cause of this syndrome is gain‐of‐function mutations in a gene encoding a potassium channel (*KCNT1*), but recently, in a cohort of 135 patients with EIMFS, two were found with de novo heterozygous pathogenic variants in *PIGA*, an X‐linked gene involved in GPI‐anchor biosynthesis, implicating this pathway in this particular epileptic syndrome.^21^ Presently, the downstream GPI‐AP target(s) involved in the pathogenesis of IGDs remain unidentified.

St1, who was homozygous for the p.(R538Afs*24) variant, seems to have the mildest phenotype in our cohort. She is currently 11 years old and although she has chewing difficulties, she remains the only living patient who is not G‐tube dependent. While she has limited fixing/tracking abilities, she is also the only patient without a cortical visual impairment. While she is homozygous for a frameshift in the last exon, it is unclear if this can explain her milder phenotype, as St6, with a more severe phenotype and who eventually died, had an even later frameshift p.(G557Dfs*4). This may be in part because St6 was born from consanguineous parents thus other recessive genes may be contributing, or there may not be a clear genotype‐phenotype correlation. Indeed, even the two siblings in our cohort (St3a and St3b) had different clinical presentations and survived to 2 days and 5 years, respectively. Analysis of further affected individuals may reveal a genotype‐phenotype correlation, and given that PIGQ is part of the N‐acetylglucosamine complex, the protein location of the disease‐causing variants may result in differential impact for the binding of other enzymes in the complex. Compared to other IGDs, there are more often bi‐allelic nonsense and/or frameshift variants in *PIGQ*, but there is also increased mortality.^22^ This might be because bi‐allelic loss‐of‐function mutations in other GPI‐biosynthesis genes lead to embryonic lethality, while it does not for *PIGQ*.

Previous studies have correlated variants in *PIGT*, another key component to GPI‐biosynthesis, to preferential atrophy of the cerebellar vermis,^18^, ^23^, ^24^, ^25^ a finding found inconsistently in a subsequent cohort.^17^ Similarly, only two of our patients (St3b and St5) had MRI results showing atrophy affecting the cerebellar vermis, however, interestingly, an MRI of St5 at age 2 years found diffuse cerebral atrophy, but cerebellar atrophy was not appreciated. Given that variants in other genes in this pathway have been linked to cerebral and cerebellar loss of tissue over time, it is worth considering further imaging later in childhood.

Although serum alkaline phosphatase (ALP) has been linked with IGDs, evidence suggests that elevated ALP levels are most often associated with variants in later steps of GPI biosynthesis, whereas earlier steps are associated with ER‐associated degradation of the anchor.^26^, ^27^ Thus, it is not surprising that elevated serum ALP is not common among subjects with *PIGQ* pathogenic variants.

Treatments remain limited, though butyrate helped treat intractable epilepsy due to variants in the *PIGM* promoter,^28^ and IGDs due to variants in *PIGV*, *PIGO*, and *PIGS* have been partially treated with vitamin B6 supplementation.^29^, ^30^, ^31^ In St6, there was phenotypic overlap with cerebral folate deficiency due to *FOLR1*‐mutations,^32^ in which the GPI‐anchored folate receptor alpha fails to provide folate to the brain. This can be by‐passed by providing folinic acid supplementation, however, unfortunately, St6 did not respond to this therapy, though it should be noted it was initiated late in his disease course.

Given the function of the proteins involved in GPI‐anchor biosynthesis, the gold standard for evaluation of functional defects in this pathway is using flow cytometry to assess the effect of the variants on GPI‐anchored proteins.^29^ In granulocytes from fresh blood and fibroblasts, we show reduced expression of GPI‐anchored proteins in five unrelated affected individuals, a phenotype that was partially or fully reversible in fibroblasts by transfecting WT cDNA. Taken together with previous work on CHO cells, we show that biallelic *PIGQ* variants impact GPI‐AP expression.

In summary, we expand the phenotypic spectrum of IGDs related to biallelic *PIGQ* pathogenic variants and show that the impaired expression of GPI‐APs in human cells can be improved in vitro by the transduction of WT *PIGQ* cDNA. These variants lead to impaired GPI‐biosynthesis and subsequently reduced localization of GPI‐APs, resulting in a broad phenotypic spectrum. While the phenotypic spectrum is broad, the clinical overlap of IGDs is significant, and collectively they may represent as much as 0.15% of all developmental disorders.^33^ Thus, there is a need for future work to develop therapies that reduce the impact of these devastating diseases.^34^

## CONFLICT OF INTEREST

Devon L. Johnstone, Thi Tuyet Mai Nguyen, Jessica Zambonin, Kristin Kernohan, Anik St‐Denis, Nissan V. Baratang, Taila Hartley, Michael T. Geraghty, Julie Richer, Jacek Majewski, Eric Bareke, Andrea Guerin, Manuela Pendziwiat, Loren D.M. Pena, Hilde M. H. Braakman, Karen W. Gripp, Andrew C. Edmondson, Miao He, Rebecca C. Spillmann, Erik A. Eklund, Allan Bayat, Hugh J.McMillan, Kym M. Boycott, and Philippe M. Campeau declare that they have no conflict of interest.

## ETHICS STATEMENT

All procedures followed were in accordance with the ethical standards of the responsible committee on human experimentation (institutional and national) and with the Helsinki Declaration of 1975, as revised in 2000.^35^ Informed consent was obtained from all patients for being included in the study.

## AUTHOR CONTRIBUTIONS

Devon L. Johnstone, exome analysis, Sanger sequencing, collection and analysis of clinical data, functional work, drafting of manuscript. Thi Tuyet Mai Nguyen, flow cytometry, experiment planning, drafting of manuscript; Jessica Zambonin, collection and analysis of patient data, revision of supplemental clinical data summaries; Kristin Kernohan, exome analysis, experiment planning, editing of manuscript; Anik St‐Denis, flow cytometry, editing of manuscript; Nissan V. Baratang, flow cytometry, editing of manuscript; Taila Hartley, exome analysis, editing of manuscript; Michael T. Geraghty, collection and analysis of patient data, editing of manuscript; Julie Richer, collection and analysis of patient data, editing of manuscript; Jacek Majewski, exome analysis, editing of manuscript; Eric Bareke, exome analysis, editing of manuscript; Andrea Guerin, collection and analysis of patient data, editing of manuscript; Manuela Pendziwiat, collection and analysis of patient data, editing of manuscript; Loren D.M. Pena, collection and analysis of patient data, editing of manuscript; Hilde M. H. Braakman, collection and analysis of patient data, editing of manuscript; Karen W. Gripp, collection and analysis of patient data, editing of manuscript; Andrew C. Edmondson, collection and analysis of patient data, functional data, editing of manuscript; Miao He, cell culture and flow cytometry, editing of manuscript; Rebecca C. Spillmann, collection and analysis of patient data, editing of manuscript; Erik A. Eklund, collection and analysis of patient data, editing of manuscript; Allan Bayat, collection and analysis of patient data, editing of manuscript; Hugh J. McMillan, collection and analysis of patient data, MRI analysis, editing of manuscript; Kym M. Boycott, co‐senior author; Philippe M. Campeau, co‐senior author.

## Supporting information




**APPENDIX** **S1**: Supporting Information
