# Supplementary material for: Early infantile epileptic encephalopathy due to biallelic pathogenic variants in PIGQ: Report of seven new subjects and review of the literature
Source: J Inherit Metab Dis. 2020 Aug 3;43(6):1321–32. doi: 10.1002/jimd.12278 (PMC7689772; doi:10.1002/jimd.12278)
Supplement: Supplementary file 1 — APPENDIX S1: Supporting Information [file JIMD-43-1321-s001.docx]

**SUPPLEMENTARY MATERIAL**

**Early infantile epileptic encephalopathy due to biallelic pathogenic variants in *PIGQ*: Report of 7 new subjects and review of the literature**

Devon L. Johnstone^[[1]](#footnote-2)^, Thi Tuyet Mai Nguyen^[[2]](#footnote-3)^, Jessica Zambonin ^1,^^[[3]](#footnote-4)^, Kristin D. Kernohan^1,4^, Anik St-Denis^2^, Nissan V. Baratang^2^, Taila Hartley^1^, Michael T. Geraghty^[[4]](#footnote-5)^, Julie Richer^3^, Jacek Majewski^[[5]](#footnote-6),^^[[6]](#footnote-7)^, Eric Bareke^5,6^, Andrea Guerin^[[7]](#footnote-8)^, Manuela Pendziwiat^[[8]](#footnote-9)^, Loren D.M. Pena^[[9]](#footnote-10),^^[[10]](#footnote-11)^, Hilde M.H. Braakman^[[11]](#footnote-12),^^[[12]](#footnote-13)^, Karen W. Gripp^[[13]](#footnote-14)^, Andrew C. Edmondson^[[14]](#footnote-15)^, Miao He^[[15]](#footnote-16)^, Rebecca C. Spillmann^[[16]](#footnote-17)^, Erik A. Eklund^[[17]](#footnote-18)^, Allan Bayat^[[18]](#footnote-19),^^[[19]](#footnote-20)^, Undiagnosed Diseases Network, Care4Rare Canada Consortium, Hugh J. McMillan^[[20]](#footnote-21)^, Kym M. Boycott^1,3,*^, Philippe M. Campeau^2,^^[[21]](#footnote-22),*^

**TABLE OF CONTENTS**

**Patient recruitment** 3

**Sequencing methods** 4

**Clinical descriptions**  7

**Figure S1** 18

**Figure S2** 19

**Table S1** 20

**Table S2** 21

**Table S3**  26

**Table S4**  27

**References**  28

**Appendix 1** 30

**PATIENT RECRUITMENT**

Subject 1 (St1) was recruited by the EuroEPINOMICS Rare Epilepsy Syndromes (RES) consortium. Subject 2 (St2) was recruited by the DuPont Hospital for Children/Nemours and the Children’s Hospital of Philadelphia. Ethics approvals were obtained from the local institution review boards and informed consent was obtained from patients´ parents.

Subject 3a, 3b and 4 (St3a, St3b, St4) were recruited as part of a Care4Rare Canada research study, which was approved by the Children’s Hospital of Eastern Ontario Research Ethics Board (REB #11/04E).

For subject 5 (St5), oversight for the human subjects research protections was provided by the institutional review board (IRB) of the National Human Genome Research Institute (protocol 15-HG-0130, Clinical and Genetic Evaluation of Patients with Undiagnosed Disorders through the Undiagnosed Diseases Network) and the Duke University School of Medicine IRB (Pro00056651).

Subject 6 (St6) was recruited as part of the Swedish MIDIC (Mitochondrial Disease in Children) study, reviewed by the local Ethical Review Board in Lund (protocol #2009/97; amended #2017/266).

**SEQUENCING METHODS**

Primers used for Sanger sequencing validations are listed in Table S1. Exome sequencing details for each patient/family are as follows:

**St1**

Exome sequencing was performed on an Illumina HiSeq2000 by BGI-Europa, after exome enrichment with the Agilent SureSelectXT Human All Exon 50Mb Kit using blood-derived DNA from the patient and both parents. Read alignment was performed with Burrows-Wheeler Aligner,^1^ and variant calling using GATK,^2^ with annotation performed by the Department of Genetics of the Radboud University Medical Center with an in-house developed program. The variants in genes were selected based on possible pathogenicity (deletions, insertions, nonsense and canonical splice site mutations and missense mutations (PhyloP score base change >3.5)). Variants were confirmed by Sanger sequencing (Table S1).

**St2**

Exome sequencing was performed on an Illumina HiSeq2000 by GeneDx, after exome enrichment with the Agilent SureSelect XT2 All Exon V4 kit using blood-derived DNA from the patient and both parents. The DNA sequence compared to human genome build GRCh37 (hg19) reference sequence. The targeted coding exons and splice junctions of known protein coding RefSeq genes were assessed. The Xome Analyzer was used to evaluate sequence changes of the proband relative to the parents. Variants were confirmed by Sanger sequencing (Table S1).

**St3a and St3b**

Exome sequencing was performed on an Illumina HiSeq2000, after exome enrichment with the Agilent SureSelect Clinical Research Exome kit using blood-derived DNA from the proband (St3b) and both parents. A pipeline based on Burrows-Wheeler Aligner,^1^ Picard (http://broadinstitute.github.io/picard/), ANNOVAR^3^ and custom annotation scripts was used. The GRCh37 (hg19) human reference genome assembly was used for alignment. Variants were compared to the 1000 genomes phase 1 data set (April 2012 release),^4^ the Exome Variant Server (http://evs.gs.washington.edu/EVS/), ExAC,^5^ and an in-house database of ~2000 exomes previously sequenced at the McGill University and Génome Québec Innovation Centre. Variants with greater than 1% frequency in any database were excluded from further analysis. Variants were confirmed by Sanger sequencing (Table S1), and DNA from the sibling, St3a, was subsequently Sanger sequenced to confirm the presence of the variants.

**St4**

Exome sequencing was performed at Prevention Genetics as per their protocol (https://www.preventiongenetics.com/ClinicalTesting/TestCategory/PGxome.php) using blood-derived DNA from patient and both parents. The GRCh37 (hg19) human reference genome assembly was used for alignment. Bam files were analyzed using SAMtools mpileup,^6^, Freebayes (arXiv preprint arXiv:1207.3907), Platypus^7^ and GATK-haplotype^8^ to identify single nucleotide variants and short insertions or deletions (indels). Variants were further analyzed if identified in >2 programs. Variants were annotated using Ensembl variant effect predictor^9^ and the ENSEMBL/Gencode gene set, and annotated with VEP using the Refseq gene set^10^ and GEMINI framework.^11^ Variants that were previously detected in more than 20 of local control samples and/or have had a minor allele frequency greater than 1% in the 1000 genomes/ ESP/ NHLBI exome database and/or those identified in one or more NHLBI exomes and had been called with a homozygous genotype were excluded. Clinical significance of the remaining variants was assessed using Alamut Visual in accordance with ACMG recommendations.^12^ All rare variants were checked for prior reports in the literature, the ClinVar, ExAC, Exome Variant Server databases and other relevant sources of information. Variants were confirmed by Sanger sequencing (Table S1).

**St5**

Exome sequencing and data analysis was performed at Baylor Genetics following their standard protocol (https://www.bcm.edu/research/medical-genetics-labs/test_detail.cfm?testcode=1600) using blood-derived DNA from the patient and both parents. Variants were confirmed by Sanger sequencing (Table S1).

**St6**

Exome sequencing was performed on an Illumina HiSeq2500, after exome enrichment with the Agilent SureSelect Clinical Research Exome kit using blood-derived DNA from the patient and mother. Bioinformatic analysis was performed using an in-house Mutation Identification Pipeline (MIP).^13^ Variants were filtered using an epilepsy panel using version six of our database (https://www.karolinska.se/for-vardgivare/karolinska-universitetslaboratoriet/centrum-for-medfodda-metabola-sjukdomar/genetisk-diagnostik/). Variants were confirmed by Sanger sequencing (Table S1).

**CLINICAL DESCRIPTIONS**

**St1**

Subject 1 was the third child to non-consanguineous healthy Turkish parents with an unremarkable family history. She was born at 38 weeks and 2 days gestation following an uncomplicated pregnancy and delivery and weighed 3420g (65.5%). She met early developmental milestones however her development plateaued at 6 months of age, coinciding with the onset of seizures. Currently, at 11 years of age, she continues to have intermittent seizures of multiple semiology including focal, tonic, bilateral tonic-clonic, with impaired awareness, but no classical absences. These tend to be clustered and often precipitated by fever. She sometimes has long seizure-free intervals (months) between episodes. She is currently treated with carbamazepine, phenobarbital and clobazam. She has a severe developmental disability; she makes few sounds and is unable to ambulate independently. She has some purposeful hand movements and can sit independently for brief periods when placed in position. She can fix and follow intermittently. She is incontinent of urine and stool and has difficulties with constipation, swallowing and sleep. She has stereotypies including, shaking of her head, closure of her eyes, rolling of her eyes, intermittent groaning and hyperventilation. She has abnormal movements including, dystonic posturing of her left foot, choreatic movements of her hand and on examination has axial hypotonia, limb hypertonia, ankle contractures and brisk deep tendon reflexes. She is non-dysmorphic and has strabismus, pectus excavatum, inverted nipples and hip dysplasia.

Exome sequencing demonstrated a homozygous *PIGQ* variant: Chr16(GRCh37):9.632962del; NM_148920.2:c.1611del, p.R538Afs*24. Clinical flow cytometric analysis confirmed that the GPI anchor is involved, with a mean fluorescence intensity (FLAER) of granulocytes of 22249 versus 34385 in the control (64.7%) (histogram not available).

**St2**

Subject 2 was the second child to a healthy non-consanguineous couple of European and Puerto Rican descent. The patient’s parents had a prior early trimester miscarriage and a healthy daughter; there was also a paternal half-sibling. The pregnancy was complicated by polyhydramnios and elevated alpha-fetoprotein (AFP) on maternal serum screening. Routine anatomy ultrasound was normal. Given the prenatal findings, an amniocentesis was performed showing normal female karyotype. She was born at 38 weeks gestation, weighing 4850g (99.9%). Issues during the neonatal period included the need for CPAP respiratory support, intermittent hypoglycemia and she failed the newborn hearing screen (left ear). Following discharge, her parent’s initial concerns were global developmental delay.

At age 7 months of age she developed focal seizures with secondary generalization. Seizures were well controlled on topiramate and levetiracetam with occasional breakthrough seizures with illness. She had a brain magnetic resonance imaging (MRI) which showed slightly diminutive frontal lobes and slightly prominent frontal horns of the lateral ventricles but was an otherwise unremarkable contrast-enhanced MRI of the brain. Magnetic resonance spectroscopy (MRS) of the brain showed a borderline lactate peak identified in nearly all the voxels placed over the basal ganglia (Table S4).

At last assessment (6 years, 6 months of age), she had chronic static encephalopathy with global developmental delay. She was G-tube dependent with chronic constipation. She had respiratory insufficiency with bilevel positive airway pressure (BPAP) dependence, neuromuscular scoliosis and pectus excavatum. She had frequent hospitalizations for respiratory illnesses leading to acute respiratory failure. During one hospitalization for respiratory infection she developed hyperkinetic involuntary movements without correlate on electroencephalogram (EEG) which resolved with clinical improvement (Table S3). She was also found to have subglottic stenosis requiring laryngeal dilation. Echocardiogram showed prolapsed mitral valve with mild left ventricular dysfunction and an electrocardiogram (ECG) showed 1st degree AV block. She is legally blind and electroretinograms (ERG) performed at 1 and 4 years of age suggested a transmission problem at the level of the photoreceptor-bipolar cell synapse in the retina. At age 3 ½ years of age she developed an episode of rhabdomyolysis.

She was evaluated by Clinical Genetics and Metabolism over several years. She was noted to have dysmorphic facial features with somewhat coarse appearance, gingival hyperplasia, midface hypoplasia, wide mouth, thickened lips, thick eyebrows and prognathism (Figure S1). Investigations were non-diagnostic including chromosomal microarray, Noonan syndrome panel, acylcarnitine profile, plasma amino acids, lactate/pyruvate ratio, carbohydrate deficient transferrin via mass spec matrix-assisted laser desorption/ionization time-of-flight (MALDI-TOF) and clinical exome sequencing. Reanalysis of clinical exome revealed two variants in *PIGQ*: NM_148920.2:c.1199_1201delACT, p.Y400del (maternal) and NM_148920.2:c.942+1G>A, IVS4+1G>A (paternal)). The average depth of coverage for the analysis was 167x, with a quality threshold of 98.8%. 100% of the coding region of the *PIGQ*gene was covered at a minimum of 10X in the XomeDx test.

Additional variants of uncertain significance reported as possibly related to the phenotype of failure to thrive, hypotonia, seizures and developmental delay, but which were clinically not thought to explain the phenotype included maternally inherited VUS in *ATP5A1* c.171G>T, p.E57D and paternally inherited VUS in *EFHC1* c.694C>T, p.L232F. In silico analysis was inconsistent in its predictions as to whether these variants are damaging to the protein structure/function.

**St3a and 3b**

Subjects 3a and 3b were sisters. Their parents were healthy, non-consanguineous and of British and French-Canadian ancestry. They had an older healthy brother and the remainder of the family history was non-contributory.

Anatomy ultrasound for Subject 3a showed prominent fetal kidneys at 26 weeks gestation. She was the product of a healthy early pregnancy later complicated by premature rupture of membranes at 28 weeks gestation, with a birth weight of 1344g. APGAR scores were 6 and 7. During the immediate neonatal period she developed respiratory distress requiring intubation and renal failure. She passed away on day 2 of life. Physical examination revealed minor dysmorphic facial features including down-slanting palpebral fissures, hypertelorism and flattened nasal bridge. Post-mortem autopsy showed tortuosity of the ureters with hypoplasia of the renal pelvis and calyces and an accessory spleen. The brain showed evidence of periventricular leukomalacia. Karyotype was normal female.

Subject 3b was born at born at term after an uncomplicated pregnancy. She weighed 3.5 kg (75%) with no resuscitation required. She developed jaundice on day two of life which responded to phototherapy. She was discharged home on day of life 3. Parents first became concerned about her development just before three months of age. She had central hypotonia, increased peripheral tone and dysconjugate gaze. She was evaluated at 4 months of age for the question of seizures however EEG was normal (Table S3) and MRI showed increased CSF spaces (Table S4). Eye examination at 5 months of age showed cortical visual impairment. During infancy, hypotonia evolved into spasticity and dystonia. She required G-tube feeds. At the age of two years she presented again with episodes concerning for seizures and EEG confirmed seizure activity in the right temporal lobe (Table S3). The seizures proved to be refractory to multiple anticonvulsants. She developed progressive microcephaly; OFC as a neonate was at the 70^th^ centile and 3^rd^ centile at the age of 4 years. She had delayed dental eruption and gingival hyperplasia but was otherwise non-dysmorphic. She developed first-degree AV block and continued to be followed for this for several years with no intervention required.

She was followed by the Genetics and Metabolics services and investigations were non-diagnostic including chromosomal microarray, enzymology for Krabbe disease, Metachromatic Leukodystrophy and Niemann-Pick A, and muscle biopsy and fibroblast studies for Pallister-Killian syndrome.

At 5 years of age she developed increasing discomfort with feeds of unknown etiology. She subsequently developed increased work of breathing and had two episodes of asystole and was unable to be revived from the second episode. Exome sequencing had been initiated prior to her passing. It revealed two variants in *PIGQ*: NM_148920.2:c.1578_1579delCC, p.Q527Afs*75, (maternal) and NM_148920.2:c.1199_1201delACT, p.Y400del (paternal). The *PIGQ* variants were confirmed by Sanger sequencing in the DNA sample from Patient 3a.

A heterozygous VUS in *PKDH1* (c.12133G>T, p.G4045W) was also identified but was not considered likely to explain the clinical presentation.

**St4**

Subject 4 was the first child to non-consanguineous Lebanese and Iraqi ancestry parents. Prenatal history included maternal Remicade exposure until 17 weeks gestational age when mother became aware of the pregnancy. Pregnancy was complicated by severe polyhydramnios, first noted at 28 weeks gestation, requiring multiple amnio reductions and severe fetal hepatomegaly (liver extending into iliac crest). Antenatal testing included a normal chromosomal microarray and no serological evidence for an intrauterine infection. Mild bilateral hydronephrosis was noted at 35 weeks gestation. She was born via uncomplicated C-section at 36+3 weeks gestation with APGARS of 8 and 9. Her birthweight was 3389 g (96%ile), birth length was 47.5cm (68.2%ile) and head circumference was 33.5 cm (84%ile). Neonatal abdominal ultrasound revealed intestinal malrotation (surgically corrected by a Ladd procedure on the first day of life). She was grossly hypotonic and significantly dysmorphic at birth with coarse facial features, increased nuchal fold, macroglossia, wide nasal bridge with a short-upturned nose, clenched hands with deep palmar and plantar creases and an impression of rhizomelic shortening. She was also noted to have hepatomegaly, a soft palate cleft and inverted nipples. She did not blink to visual threat however ophthalmological examination was structurally normal. Deep tendon reflexes were diffusely brisk (3+) and her appendicular tone was increased with episodes of extensor posturing of her upper extremities noted. An awake EEG revealed episodic slowing of background cerebral activity with intermittent asynchrony (Table S3). Several of the posturing episodes were captured which were not associated with any electrographic changes noted; felt to be clinically consistent with dystonia. MRI of the brain revealed no intracranial abnormalities (Table S4). Echocardiogram was reassuring although she had significant arrhythmic episodes in the first week of life.

Baseline investigations including serum creatine kinase, lactate alanine aminotransferase (ALT), acylcarnitine profiles were normal. Very long chain fatty acids (VLCFA) were normal. Enzyme (WBC) testing for beta-glucosidase, beta-galactosidase were negative. Plasma amino acids and urine organic acids were normal.

Due to feeding issues and failure-to-thrive, a gastrostomy tube was placed at 6 weeks old. She was re-admitted to hospital at 3 months old with *Klebsiella* pneumonia. Supplemental oxygen was required from which she could never be fully weaned. Central apneas were noted during sleep and repeat echocardiogram at 3 months’ old noted mild right ventricular hypertrophy and pulmonary hypertension. Repeat awake EEG at 4 months old showed generalized slowing of background activity (Table S3).

Over subsequent months, she demonstrated no neurodevelopmental gains. At 6 months old she demonstrated no head control and no social smile. Her eye movements were dysconjugate and she was unable to visually fixate or track consistent with cortical blindness. Her hands were closed at rest. Her head circumference was 41.0 cm (10%). Appendicular tone was increased with brisk reflexes and sustained ankle clonus.

Repeat MRI brain at 7 months old revealed progressive cortical and subcortical volume loss (Figure 2, Table S4) with a mild delay in myelination reported. Restricted diffusion was seen in the brainstem (Figure 2) corresponding to the location of the medial lemniscus tracts, bilaterally. No restricted diffusion was apparent elsewhere and MR spectroscopy was normal. Evoked potentials were performed at 7 months old. Visual evoked potentials revealed no cortical responses. Brainstem auditory evoked potentials revealed no response on the right and low amplitude and prolonged absolute and interpeak latencies on the left. EEG at 7 months old revealed hypsarrhythmia and bursts of generalized polyspike and wave discharges (Table S3). Several decremental events were captured which were clinically associated with subtle shoulder elevation; clinically consistent with epileptic spasms. Vigabatrin therapy was initiated. Within 2 weeks she was admitted to the pediatric intensive care unit (PICU) with episodes of autonomic dysfunction characterized by intermittent bradycardia, apneic episodes, hypoxia and hypothermia requiring intubation. No viral or bacterial infection could be identified. Muscle biopsy was performed during this admission which was unrevealing. mtDNA sequencing revealed no pathogenic variants. During her first week of ICU admission she developed an episode of sustained ventricular tachycardia treated with amiodarone. Repeat echocardiogram confirmed good biventricular function. Once a genetic diagnosis was made, her parents requested palliation and she died at 9 months of age.

Following unrevealing clinical exome sequencing, a re-analysis was performed under the Care4Rare Canada research protocol and revealed compound heterozygous mutations in *PIGQ*: NM_148920.2:c.1130_1168del, p.A377_S389del (maternal) and NM_148920.2:c.1345G>C, p.G449R (paternal).

**St5**

Subject 5 was the fifth pregnancy to healthy, non-consanguineous parents of Mexican ancestry. The couple had a history of a son who died shortly after birth, at 9 hours of life. He was born via C-section due to being large for gestational age and had abdominal distention and brain abnormalities noted birth; however, details are limited. The couple also had two healthy daughters. The patient was born following a pregnancy that was complicated by abnormal ultrasound findings during the second trimester that included right ventricular hypertrophy, lateral ventricles at 8mm with dangling choroids, hypoplastic inferior cerebellar vermis, leading to impression of a Dandy Walker variant, bilateral urinary tract dilation and ambiguous genitalia. He was born via a C-section at 37 weeks due to being large for gestational age and weighed 4519g (98.5%) at birth with normal APGAR scores. He spent 13 days in the NICU due to respiratory distress, jaundice and hypoglycemia. During his stay in the NICU, he was diagnosed with a patent ductus arteriosus (PDA), patent foramen ovale (PFO), mild right ventricular hypertrophy, and bilateral hydronephrosis with reflux and a connection of the distal ureters to the posterior urethra.

Concerns about his development began shortly after birth and currently at the age of 26 months he has not made neurodevelopmental gains. He has no gross or fine motor skills and is unable to smile, laugh or track objects. He was found to have cortical visual impairment at 10 months of age. Initial EEG and brain MRI were normal. However repeat EEG in infancy showed multifocal spikes and sharp waves with background slowing consistent with epileptic potential (Table S3). He has multiple seizures daily. A repeat brain MRI at 24 month showed abnormal myelination characterized by increased T2 signal in the peripheral and periatrial white matter, pituitary hypoplasia, diffuse cerebral volume loss, and abnormal skull shape with right plagiocephaly (Table S4). He experiences abdominal distension and feeding intolerance that required G-tube placement.

Previous nondiagnostic testing included plasma amino acids, urine organic acids, total and free carnitine, acylcarnitine profile, urine MPS screen, 7-dehydrocholesterol, BWS methylation testing, VLCFA, karyotype, Fragile X, and chromosomal microarray.

He was enrolled in the Undiagnosed Diseases Network (UDN) at 23 months of age. On exam he was found to have dysmorphic features including course facies, large ears, and open mouth with protruding, large tongue. He has no meaningful social interactions, his hands were primarily fisted, did not fix/track, and had profound axial and limb hypotonia with significant head lag. Trio whole exome sequencing through the UDN identified two variants in trans in the *PIGQ* gene: NM_148920.2:c.49G>A, p.G17R (maternal) and NM_148920.2:c.942+1G>A, IVS4+1G>A (paternal).

VUSs included a maternally inherited heterozygous variant in *KMT2D* (c.15736A>G, p.I5246V), a paternally inherited heterozygous variant in *LZTR1* (c.370G>A, p.V1241), and he was compound heterozygous for the variants c.868A>G, p.S290G (maternal) and c.2891C>A, p.T964N (paternal) in *ASTN1*. These variants were ruled out due to phenotype incompatibility, benign predicted pathogenicity or inheritance.

**St6**

Subject 6 was the only child to a consanguineous Afghani couple (first-cousin union). The pregnancy was unremarkable with routine prenatal care. The delivery was complicated by failure to progress requiring vacuum-assist at term. He was born at 40+1 weeks, with a birthweight of 2.94 kg (19.3%). APGAR scores were 8 and 9. There were no immediate neonatal concerns and he was discharged home. During the first week of life, he developed feeding difficulties and irritability and at 3 weeks of age he developed opisthotonos when crying, central hypotonia, appendicular hypertonia and spasticity with clenched hands. He had coarse facial features but was otherwise non-dysmorphic. An MRI scan completed at the time was normal (Table S4). Metabolic investigations including ammonia, lactate, plasma and urine amino, urine organic acids, acylcarnitine profile, water soluble vitamins, glycoconjugates in urine and cerebrospinal fluid analysis (cells, lactate, pyruvate, amino acids, protein, albumin) were all normal.

At 2.5 months he was hospitalized after a suspected seizure with eye deviation towards the upper left corner and an increased tone in all extremities. EEG at this time was normal and as was a repeat MRI of the brain (Tables S3, S4). He thereafter showed a profound stagnation of development making no meaningful gains. His clinical seizures continued and at 6 months of age an EEG showed asymmetry with sharp-waves on the left hemisphere and rhythmic sequences up to 30 seconds in keeping with electrographic seizures (Table S3). He was started on phenobarbital, topiramate and levetiracetam with initial improvement. Seizures re-occurred again at one year of age with focal seizures from both hemispheres and the EEG, consistent with the diagnosis of epilepsy of infancy with migrating focal seizures (Coppola-Dulac syndrome) (Table S3). At this time he also had a G-tube inserted for feeding. Benzodiazepines and valproate were added in conjunction to his other anti-epileptics; however, from the age of 1-2.5 years seizures were refractory. He has repeated respiratory infections leading to chronic respiratory insufficiency and at the age of 3 years and 9 months, he died from complications secondary to pneumonia. Autopsy was declined.

Exome sequencing of the patient and his mother revealed a homozygous *PIGQ* variant: NM_148920.2:c.1670del, p.G557Dfs*4. The average depth of sequencing was 330X for the patient and 263X for the mother.

Only one other mutation of potential significance was noted after the filtering process. This was a heterozygous mutation in *SPTAN-1* (c.6503C>T, p.A2168V), which was benign by PolyPhen2 and exists in low frequency in population databases. As only the mother’s DNA was available, it is not known whether this variant is *de novo* or inherited from the father.


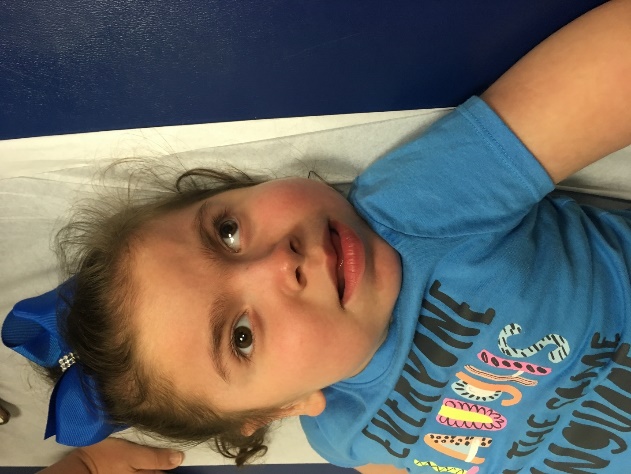

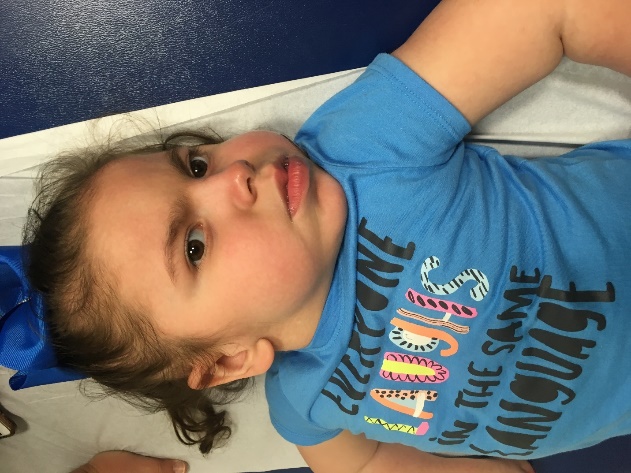

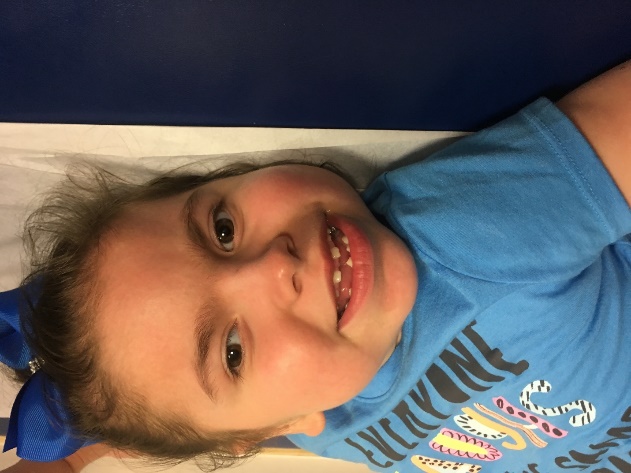


**FIGURE S1** Photographs of patient, St2, showing dysmorphic facial features with somewhat coarse appearance, midface hypoplasia, wide mouth, thickened lips, thick eyebrows and prognathism.


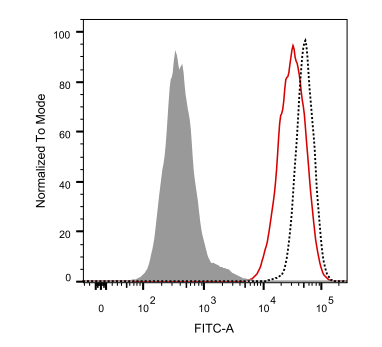


**FIGURE S2** Flow cytometry histogram of FITC-CD59 stained fibroblasts from St2 (red) relative to control (blue), grey is unstained control.

**PRIMER INFORMATION**

**Table S1** Primers used for Sanger sequencing confirmation of exome sequencing results

| Variant | Forward Primer (5’-3’) | Reverse Primer (5’-3’) |
| --- | --- | --- |
| c.1611del; p.R538Afs*24 | ATGTCTGAGACAGCACTGGC | CCAGCAGTTCCCTCAGTCCT |
| c.1578_1579del; p.Q527Afs*75 | GAGCTCAGACCACCCCACT | TTCCCTCAGTCCTGCTTGTC |
| c.1199_1201del; p.Y400del | CTTCGTGGAGCACATCCTTT | CGCAGAACGTTCCACTTCTT |
| c.1130_1168del; p.A377_S389del | CTGTGTGTGTGAGGGTTGTG | CCCACCTGGCTCCATAGAC |
| c.1345G>C; p.G449R | CTGCTACTGGGACTGCTTCT | GTGCTCAGCTCACCAGGG |
| c.942+1G>A; IVS4+1G>A | CCTTCTCCATCCCCCTCTG | CCTGGAGTTCCTGCTTCTGG |
| c.49G>A; p.G17R | AGCCGAGCCTCTCCTCTTCT | CTGCGGAAGAGCACCTCACT |
| c.1670del; p.G557Dfs*4 | Not available | Not available |

**Table S2** Detailed clinical features of seven novel patients from six families with biallelic variants in PIGQ and review of the literature

| Subject ID/Source | St1 (this report) | St2 (this report) | St3a (this report) | St3b (this report) | St4 (this report) | St5 (this report) | St6 (this report) | Martin et al 2014^14^ | Alazami et al 2015^15^ | Starr et al 2018^16^ |
| --- | --- | --- | --- | --- | --- | --- | --- | --- | --- | --- |
| Variants & Inheritance  (NM_148920.2) | Homozygous:  c.1611del  p.R538Afs*24 | Maternal  c.1199_1201del  p.Y400del  Paternal:  c.942+1G>A  IVS4+1G>A | Maternal:  c.1578_1579del  p.Q527AfsX75  Paternal:  c.1199_1201del  p.Y400del | Maternal:  c.1578_1579del p.Q527AfsX75  Paternal:  c.1199_1201del  p.Y400del | Maternal:  c.1130_1168del p.A377_S389del  Paternal  c.1345G>C  p.G449R | Maternal:  c.49G>A  p.G17R  Paternal:  c.942+1G>A  IVS4+1 G>A | Homozygous:  c.1670del  p.G557Dfs*4 | Homozygous:  c.690-2A>G | NM_004204.3:  c.619C>T  p.R207* | Maternal:  c.968_969del  p.L323Pfs*119  Paternal:  c.1199_1201del  p.Y400del |
| Gender | Female | Female | Female | Female | Female | Male | Male | Male | N/A | Male |
| Ancestry | Turkish | European/Puerto Rican | British Isles/French Canadian | British Isles/French Canadian | Lebanese/Iraqi | Mexican | Afghani | West African | N/A | N/A |
| Consanguinity? | No | No | No | No | No | No | Yes, cousins | Reported no, but extensive regions of homozygosity | N/A | No |
| History of miscarriages | Yes 1 | 1 early trimester loss | No | No | No | Yes 1 | No | N/A | N/A | N/A |
| Affected siblings | None | None | St3b | St3a | None | Yes, older brother who passed away at 9 hours old | None | None | N/A | None |
| Unaffected siblings | 2 older, healthy brothers | Full sister and paternal half- sister | 11 year old sibling healthy | 11 year old sibling healthy | None | 2 healthy older sisters | None | 2 | N/A | 2 |
| Prenatal issues or findings | None | Elevated AFP on maternal serum screening, polyhydramnios, amniocentesis performed with normal 46,XX karyotype. | U/S 26 weeks showed prominent kidneys. Premature rupture of membranes and delivery at 28 weeks. | None | Severe fetal hepatomegaly (to iliac crest) and hydronephrosis at 29+6 weeks gestation; maternal Infliximab use until 17 weeks gestation. Severe polyhydramnios. | Prenatal ultrasound during second trimester noted RVH, and lateral ventricles at 8mm with dangling choroids, hypoplastic cerebellar vermis, inferiorly leading to impression of Dandy Walker Variant, bilateral urinary tract dilation, and ambiguous genitalia. Also noted to be LGA. | None | None reported | N/A | Severe polyhydramnios requiring multiple amniocentesis fluid reduction procedures. |
| Polyhydramnios | - | + | - | - | Severe | - | - | - | N/A | Severe |
| Gestational period | 38 weeks 2/7 days | 38 weeks 0/7 days | 28 weeks 1/7 days | 41 weeks 4/7 days | 36 weeks 3/7 days | 37 weeks 3/7 days | 40 weeks 1/7 day | 41 weeks | N/A | 39 weeks (C-section) |
| APGAR scores | Normal | Unavailable | 6 and 7 | 9 and 9 | 7 and 9 | 8 and 8 | 8 and 9 | N/A | N/A | N/A |
| Neonatal complications | None | Required CPAP respiratory support, hypoglycemia, failed newborn hearing screen in left ear. | Significant respiratory distress requiring intubation and ventilation. Cardiac failure with subsequent renal failure and death at 2 days of life. | Admitted to hospital for 3 days for hyperbilirubinemia. Secundum atrial ventricular defect detected after birth. | Received antenatal steroids 2-24 hours prior to delivery. Was holding breath, given CPAP at 1 minute | Feeding difficulties, respiratory distress, jaundice, PDA/PFO. | Feeding difficulties, irritability, extremity hypertonia. | At 4 weeks: cyanotic episodes with eye twitching, brief stiffening of upper body. | N/A | Feeding difficulties, trembling episodes. |
| Birth weight | 3420 g (65.5%) | 4.85 kg (99.9%) | 1344 g (91.5% corrected) | 3560 g (75.6%) | 3389 g (96%) | 4519 g (98.5%) | 2.94 kg (19.3%) | 3.83 kg (82.8%) | N/A | 3.6 kg (69.5%) |
| Birth height | N/A | 52 cm | 38.5 cm (71.2% corrected) | 51 cm (84%) | 47.5 cm (68.2%) | 50.8 cm (68.6%) | 46 cm (2%) | N/A | N/A | N/A |
| Birth head circumference | N/A | unknown | 26.5 cm (68.1% corrected) | 34.5 cm (70%) | 33.5 cm (84.1%) | 36 cm (88.7%) | 33.3 cm (18%) | 32.5 cm (6.1%) | N/A | N/A |
| Age at last follow-up | 8 years | 6 years, 6 months | 2 days | 4 years 4 months | 8 months (passed away) | 2 years 2 months | 3 year 7 months (deceased) | 2 years | N/A | 10 months |
| Weight | 24 kg (39.7%) | 18.4 kg (16.4%) | 1460 g | 14.4 kg (13.3%) | 5.39 kg (<0.1%) | 11.4kg (20.2%) | 18.4 kg (91.2%) | N/A | N/A | 7.8 kg (5%) |
| Height | 125 cm (39.4%) | 109 cm (4.5%) | 38.5 cm | 88 cm (<0.1%) | N/A | 81 cm (0.7%) | 105.5 cm (89.5%) | N/A | N/A | 69 cm (3%) |
| Head circumference | 51.5 cm | 50 cm | 26.5 cm | 47 cm (3.6%) | N/A | 46 cm (3.4%) | 46 cm (0.3%) | N/A (0.4^th^ centile at 14 months) | N/A | 45cm (37.2%) |
| Current age | 11 years | 6 years 6 months | Deceased 2d | Deceased 5y | Deceased 9m | 2y2m | Deceased 3y9m | Deceased 2y4m | N/A | Deceased 10m |
| Developmental delay | Severe DD/ID (makes sounds, does not understand simple tasks, grabs objects and brings them to her other hand, is able to sit for a short time, able to roll to her flank, not able to walk). | Severe DD/ID – chronic static encephalopathy | N/A (death at 2 days) | Global developmental delay, no walking, sitting or rolling over, very little head control. | Severe DD/ID, failure to thrive. | Profound global developmental delays (does not have head control, sit up, hands clenched, does not track or fix). | Profound global ID (no track or fix, no language, no head control, cannot sit or roll). | Profound developmental delay, lack of head control, does not smile. | Developmental delay | Lack of head control, episodes of gasping with lip quivering. |
| Seizures? | Yes | Yes | N/A | Yes | Yes | Yes | Yes | Yes | Yes | Yes |
| Seizure onset | 6 months | 7 months | N/A | Almost 4 months | 7 months | 6 months | 2.5 months | 4 weeks | N/A | 7 months |
| Seizure types | Tonic seizures and seizures during which she turns her eyes upwards and does not react to her surrounding | Focal status epilepticus, generalized tonic-clonic | N/A | Myoclonus. hand extends, full body shifting, nystagmus and 4 limb shakes. | Epileptic spasms, status epilepticus | Myoclonic jerks and generalized tonic clonic seizures. | Initially tonic seizures with eye deviation. Then status epilepticus. Thereafter epilepsy of infancy with migrating focal seizures. | Focal, tonic, tonic-clonic, cyanotics spells | N/A | Clustered myoclonic jerks with extremity stiffening, lip quivering, back arching with upper extremities appearing floppy, status epilepticus |
| Seizure control | Seizures are clustered, mostly during episodes of fever. In between a period of several months without seizures (from the outside). | Controlled on medication, breakthroughs with illness | N/A | No seizures 4 months>2 years. Thereafter, seizures occurred in cycles: 1-3 daily over 10 to 14 days, then increased to at least 8 per day then tapered off. She would then be seizure free for 7 to 12 days and the cycle would restart. Seizures would occur mainly around transition to sleep/wake. | Poor | 10 clusters of myoclonic jerks per day more with illness. Other seizure types vary (from days to months). | Started AED at 6 months. Initially well controlled with topiramate, levetiracetam and phenobarbital. Then daily migrating focal seizures with varying frequency. | Poor | Intractable | Refractory to Levetiracetam, controlled on Fosphenytoin |
| Seizure current medication | Carbamazepine, phenobarbital, clobazam | Topamax and Keppra | Deceased | Deceased | Deceased | Keppra, Onfi, Vimpat | Deceased | Deceased | N/A | Deceased |
| Seizure drugs tried (AEDs) | Carbamazepine, valproic acid, phenobarbital, clobazam | Topamax and Keppra | N/A | Carbamazepine, valproate (initial response), topiramate (no response), lamotrigine (little response), nitrazepam (some response), oxcarbazepine (some response), clobazam | Vigabatrin, Lorazepam, midazolam, phenobarbital | N/A | Levetiracetam  Topiramate  Phenobarbital  Clonazepam  Valproate  Lorazepam | Diazepam  Phenytoin  Phenbarbital  Paraldehyde  Vigabatrin  Valproic acid  Topiramate  Clonazepam | N/A | Levetiracetam  Lorazepam  Fosphenytoin  Phenobarbitol  Gabapentin |
| Other medications | Midazolam nasal spray when seizures last > 5 minutes, no other medications | Albuterol, cetirizine, enalapril, fluticasone, polyethylene glycol, simethicone, vitamin D3 | Nitrous oxide for cardiac failure | Vitamin D, ranitidine, PEG3350 | Trimethoprim, Lansoprazole, Vitamin D 400U daily, Lactulose 5ml BID | Diastat as needed | Sildenafil (pulmonary hypertension)  Furosemide  Ventolin  Budesonide  Diazepam (rectal) | Biotin  Pyridoxine | N/A | Clonidine |
| Hypotonia | Axial hypotonia, hypertonia of arms and legs. | Muscle tone axial and appendicular hypotonia with severe head lag. | N/A | Axial hypotonia, with appendicular hypertonia and mild spasticity. | Axial hypotonia, appendicular spasticity, tonic posturing of upper limbs. | Axial and appendicular hypotonia with significant head lag. | Pronounced axial hypotonia and appendicular hypertonia with clenched fists. Significant head lag | Yes | N/A | Progressive truncal hypotonia |
| Other neurological findings | Stereotypic movements (turning of her head, closure of her eyes, rolling of her eyes, intermittent groaning/hyperventilation).  strabismus convergence, dystonic positioning of her left foot, choreatic movements of her hands  Hyperlaxicity  Sleep disturbances – difficulties to falling asleep. | hyperkinetic involuntary movements. | Autopsy: normal meninges, cranial nerves and vessels. Cerebral hemispheres were normal and no evidence of malformation. Sections of the cerebral cortex show pyknotic nuclei, demonstrating karyorrhexis. There were numerous changes within the white matter. There were areas of devitalization of white matter with necrosis and karyorrhexis of cells. There are astrocytes with enlarged nuclei in these areas. The GFAP was strongly positive. This is indicative of periventricular leukomalacia. No striking anoxic changes. | Random myoclonic jerks throughout the day. | Opisthotonos | Involuntary movements of all 4 extremities, sleep disturbances, nystagmus. | Opisthotonos in the infancy  Suspected blindness, uncoordinated eye movements. | Dystonic movements | N/A | Central apnea, decreased deep tendon reflexes (1+) |
| Facial dysmorphism | No | Dysmorphic facial features with somewhat coarse appearance, midface hypoplasia, wide mouth, thickened lips, thick eyebrows, prognathism. | Small chin, macroglossia, flat nasal bridge, down slanting palpebral fissures. | High arched palate | Coarse facial features, thick lips, macroglossia, short neck, prominent forehead, wide nasal bridge and short upturned nose, large ears, micrognathia. | Coarse facial features | Coarse facial features | Thick lips, thick alveolar margins, narrow palate. | N/A | Coarse and dysmorphic facial features, hooded upper eyelids with mild ptosis, telecanthus, fleshy and uplifted earlobes, thick alae nasi with broad nasal tip, anteverted nares. Full almost pendulous cheeks, long and smooth philtrum, thin vermillion of upper lip, downturned corners of mouth, mild micrognathia. |
| Cranial shape anomalies | - | Mild brachycephaly | - | - | Cleft soft palate and high arched hard palate, progressive microcephaly (90^th^% at birth which had dropped to the 10^th^ centile by age 7 months). | Plagiocephaly, small maxilla, mild brachycephaly. | Positional plagiocephaly | - | N/A | Large anterior fontanelle, sphenoid wing dysplasia. |
| Craniosynostosis | - | - | - | - | - | Possible premature closure of the right coronal suture, the left is probably open. | - | - | N/A | Intrasutural bone of the coronal suture |
| Pectus excavatum | + | + | - | + | - | - | - | - | N/A | + |
| Other skeletal anomalies | - | Neuromuscular S-shaped scoliosis | - | - | Short stature, hyperextensive interphalangeal joints. | - | - | - | N/A | Progressive scoliosis, metaphyseal radiolucent lesions in femora and tibiae |
| Other dysmorphic features | Inverted nipples | - | Bilateral pulmonary interstitial emphysema, accessory spleen | - | Increased nuchal redundancy, deep palmar and plantar creases. | Subjectively large pinna | - | Inverted nipples | N/A | Deep plantar creases, marked abdominal laxity, prune-belly-like abdomen |
| Deafness | - | - | N/A | - | Mild left conductive hearing deficit. | - | - | - | N/A | - |
| Ophthalmological anomalies | Short fix/track | Dysconjugate gaze, cortical visual impairment Synaptic, midretinal disease with fundoscopically normal looking fundi, no optic nerve atrophy  ERG (performed at 1 year and 4 years of age) suggests that there is a transmission problem at the level of the photoreceptor-bipolar cell synapse in the retina | N/A | Cortical visual impairment | Cortical blindness, strabismus exotropia, Lagophthalmos of approximately 3-4mm of each eye, reaction to light with faint blinking, corneal ulcer of right eye. | Cortical visual impairment, unable to fix/track, nystagmus. | Cortical visual impairment, unable to fix/track. | Poor vision | Optic atrophy | Vertical nystagmus, bilateral hyperopia with astigmatism, cortical visual impairment, delayed visual maturation, punctate keratitis of both eyes, alacrima with decrease of corneal sensation. |
| Cardiac anomalies | - | Prolapsed mitral valve with mild left ventricular dysfunction, 1st degree AV block. | Right ventricular hypertrophy and poor heart function. Hypertrophy not supported on autopsy exam. | Holter monitor revealed first and second-degree heart block with some complete heart block and vestibular escape beat. | Arrhythmia | PDA, PFO (both resolved) mild pulmonary stenosis. | Tricuspid insufficiency  Pulmonary hypertension | - | N/A | Patent foramen ovale discovered at 17 weeks old. |
| Genito-urinary malformation | Incontinence | Incontinence | Dilated and tortuous ureters, hypoplastic renal pelvis, hypoplastic renal calyces. | None noted- no ultrasound done | AUS at 8 months: bilateral grade 4 hydronephrosis, new renal stones. Kidneys slightly increase in size. | Incontinence, vesico-ureteral reflux, and bilateral hydronephrosis. | Incontinence | - | N/A | Bilateral cystic renal dysplasia, bilateral inguinal hernias, small right kidney with increased cortical echogenicity and diminished cortical medullary differentiation, grade 3 vesicoureteral reflux on the right, 1 on the left. |
| Gastrointestinal issues | Constipation, chewing difficulties, incontinence. | Constipation, reflux, G-tube dependent. | - | Recurrent aspirations, G-tube. | G-tube at 6 weeks, Volvulus (Ladd’s procedure performed), SMA/SMV reversal, constipation, GERD. | G-tube dependent, duodenal web s/p resection, malrotation s/p Ladd procedure, intestinal dysmotility, volvulus, dysphagia. | G-tube dependent, constipation. | Recurrent vomiting, G-tube dependent (feeding difficulties). | N/A | Cyclic vomiting, hepatic nodule, poor feeding, diastasis recti. |
| Nephrocalcinosis | - | - | - | - | - | - | - | - | N/A | + |
| Teeth anomalies | Gingival hyperplasia | Small appearing teeth with delayed dentition. | Not applicable | Delayed eruption with gingival hyperplasia. | Too young | No teeth (delated dentition) | N/A | - | N/A | Too young |
| Nail anomalies | - | - | - | - | - | - | - | - | N/A | - |
| Short fingers or hands | - | - | - | - | - | - | - | - | N/A | - |
| Joint contractures | Yes, of ankles. Her hips are out of the hip-bowls | - | - | Mild spasticity of the lower extremities requiring AFOs | + |  | - | - | N/A | - |
| Serum alkaline phosphatase | 194 U/l (age 1.5 year), normal range 100-310 U/L | Intermittently elevated (range 212 – 1,360), normal range 150-380 U/L | Not done | 762 (2yrs), 486 (5yrs), normal range 176-530 U/L | 255-533, normal range 110-320 U/L | Not measured | Normal  At 2 yrs 5 months 330 (42-408); at 3 yrs 2 months 258. | N/A | N/A | 480-836U/L (150-440 U/L) |
| Other |  | Repeated pneumonia, chronic respiratory insufficiency, Subglottic stenosis requiring multiple episodes of Microlaryngoscopy & Bronchoscopy (MLB) with laryngeal dilation, thoracic insufficiency, asthma, Nocturnal BIPAP dependence, one episode of rhabdomyolysis. |  |  | Coarse cry, Sandifer syndrome. Failure to thrive. Abnormal control of respiratory rate. Lack of interaction. Pneumonia. |  | Repeated pneumonias. Respiratory insufficiency.  Died from pneumonia + worsening in pulmonary hypertension. | Aspiration pneumonia |  |  |

**Abbreviations:** GFAP (glial fibrillary acidic protein), N/A (not applicable or not available), PDA (patent ductus arteriosus), PFO (patent foramen ovale), centiles expressed according to WHO scale and corrected for prematurity where applicable.

**Table S3** EEG findings for each patient reported here and from the literature

| Subject | EEG findings |
| --- | --- |
| St1 | (8 years): Slow, encephalopathic, no occipital rhythm, no reaction on eye closure and opening of her eyes.  During wake in 1-10% of time multifocal epileptiform discharges, mainly left temporal/fronto-temporal.  During sleep still a slight increase in epileptiform discharges to 10-50% of time. After awakening a slight decrease to 1-10%  No seizures. During the moments of decreased consciousness during wakefulness there are no epileptiform discharges. Conclusion: localization related epilepsy. |
| St2 | (5 years): Overall Final Impression: Abnormal. This is an abnormal EEG during wakefulness and sleep due to: - A poorly organized and diffusely slow background without the expected features of wakefulness - Intermittent high amplitude left posterior slowing - Intermittent right posterior slowing with admixed right parietal sharp waves - Frequent right frontal or right parietal sharp waves that are sleep potentiated - Occasional left frontal and left parietal sharp waves that are sleep activated.  - This EEG suggests diffuse cerebral dysfunction.  There is frequent intermittent polymorphic high-amplitude 2-3 Hz delta slowing in the left temporal parietal region. There is intermittent polymorphic, semi rhythmic, moderate amplitude 3 Hz slowing in the right posterior quadrant with embedded P4 sharp waves. Sporadic Epileptiform Discharges: Type # 1: Focal. Morphology: Sharp.  Qualification: Frequent.  Location: Independent sharp waves at F4, and P4 which are present during wakefulness and potentiated by sleep. In sleep, F4 sharps occur in runs of 3-10 seconds.. Type # 2: Focal. Morphology: Sharp. Qualification: Occasional. Location: Independent sharp waves at F3, P3 which are seen only in sleep; there are rare F3 runs up to 3-5 seconds. |
| St3a | - |
| St3b | (5 months): Normal, repeat showed mild slow waves over left temporal lobe.  (2 years): 3 events localized to L posterior head region. Interictally some left posterior head spike and slow wave complexes, some sharp waves in both central head regions.  (3 years): Generalized slow wave excess out of keeping with subject age. Single right posterior temporal lobe spike. |
| St4 | (Neonatal): Negative for seizures but shows episodic slowing of background activity and asynchrony.  (8 months; admitted with epileptic spasms): Confirmed hypsarrhythmia. |
| St5 | (6 months): 4-5 hz, multifocal spikes intermittently at T5, F8 and T4.  (8 months): Generalized slowing with multifocal sharp waves and excessive beta activity no seizures.  (14 months): EEG with LTM noted abundant sleep activated multifocal spikes and sharp waves with background slowing. |
| St6 | (7 weeks): Within normal boundaries.  (4 months): Normal.  (6 months): Asymmetric EEG with slowing left side. Sharp-waves left side with short rhythmic sequences up to 30 seconds.  (14 months): Full EEG + video monitoring 24 hours. Continuous background. Repeating 2-3 min long focal seizures left and right side independently (EIMFS). |
| Martin et al 2014^14^ | (3 months): Burst-suppression pattern with bursts of high amplitude multifocal, irregular sharp and slow wave discharge, interrupted by 1-1.5 seconds of flattening in wake/drowsiness when posterior discharges were prominent, subtle focal seizure.  (2 years): Very abnormal but more continuous in wake and sleep over right hemisphere, with very active multifocal sharp waves on left side, left discharge interrupted in sleep by brief periods of EEG attenuation over the left hemisphere. Left temporal focal seizure. |
| Alazami et al 2015^15^ | N/A |
| Starr et al 2018^16^ | (2 months): Unremarkable, but clinical episodes not captured during recording.  (7 months): EEG1 frequent epileptiform discharges arising from right temporal, central, and bilateral occipital areas; EEG 2 high amplitude diffuse background slowing.  (9 months): Multifocal epileptiform activity in the form of high amplitude spike and slow wave complexes in the right occipital region, spikes and polyspikes associated with slow potentials in bilateral temporal and posterior temporal regions. |

**Table S4** MRI findings for each subject for each patient reported here and from the literature

| Subject | MRI findings |
| --- | --- |
| St1 | (8 months): Broad periventricular cerebral fluid spaces, almost no myelination to the central areas and no myelination to the occipital area. No abnormalities of the cerebral cortex. |
| St2 | (5 months): Slightly diminutive frontal lobes and slightly prominent frontal horns of the lateral ventricles. Otherwise, unremarkable contrast-enhanced MRI of the brain.  MRS: There is borderline lactate peak identified in nearly all the voxels placed over the basal ganglia. The minimal lactate peak is also seen in voxels overly CSF and the ventricles. This is nonspecific marker of parenchymal injury. The major metabolites are otherwise normal for age.  No repeat MRI obtained. |
| St3a | No imaging. |
| St3b | (19 months): Mild volume loss involving the cerebellum, particularly the vermis. There remain areas of incomplete myelination in the subcortical white matter of the bilateral frontal lobes, anterior temporal lobes, subinsular white matter and periventricular white matter around the frontal horns, atria and occipital horns of the lateral ventricles. Mild prominence of the extra-axial subarachnoid space overlying the bilateral frontal convexities, right greater than left and in the intrahemispheric fissure. |
| St4 | (9 days): Normal but spine images show low lying conus medullaris.  (7 months): Nonspecific increased T2/FLAIR intensity in bilateral medial lemniscus tracts. Prominent extra axial spaces and ventricles, showing progressive cortical volume loss and loss of subcortical white matter volume compared to prior study. Slightly delayed myelination. |
| St5 | Prenatally, lateral ventricles at 8mm with dangling choroids, hypoplastic cerebellar vermis, inferiorly leading to impression of Dandy Walker Variant.  (2 years):  1. Abnormal myelination characterized by increased T2 signal in the peripheral and periatrial white matter, but with more age-appropriate T2 signal in the deep white matter. This is favored to represent delayed  myelination. The basal ganglia and cerebellum are normal.  2. Pituitary hypoplasia with the preservation of the pituitary stalk.  3. Diffuse cerebral volume loss.  4. Abnormal skull shape with right plagiocephaly, possibly positional plagiocephaly. |
| St6 | MRI at 6 weeks and 3 months normal. |
| Martin et al 2014^14^ | (3 months): Normal.  (9 months): Delayed/limited myelination but no gross structural lesion. |
| Alazami et al 2015^15^ | N/A |
| Starr et al 2018^16^ | (5 months+10 months CT): Plagiocephaly with ventriculomegaly (left>right). |

**REFERENCES**

1. Li H, Durbin R. Fast and accurate short read alignment with Burrows-Wheeler transform. *Bioinformatics.* 2009;25(14):1754-1760.

2. McKenna A, Hanna M, Banks E, et al. The Genome Analysis Toolkit: a MapReduce framework for analyzing next-generation DNA sequencing data. *Genome Res.* 2010;20(9):1297-1303.

3. Wang K, Li M, Hakonarson H. ANNOVAR: functional annotation of genetic variants from high-throughput sequencing data. *Nucleic Acids Res.* 2010;38(16):e164.

4. Genomes Project C, Auton A, Brooks LD, et al. A global reference for human genetic variation. *Nature.* 2015;526(7571):68-74.

5. Karczewski KJ, Weisburd B, Thomas B, et al. The ExAC browser: displaying reference data information from over 60 000 exomes. *Nucleic Acids Res.* 2017;45(D1):D840-D845.

6. Li H, Handsaker B, Wysoker A, et al. The Sequence Alignment/Map format and SAMtools. *Bioinformatics.* 2009;25(16):2078-2079.

7. Rimmer A, Phan H, Mathieson I, et al. Integrating mapping-, assembly- and haplotype-based approaches for calling variants in clinical sequencing applications. *Nat Genet.* 2014;46(8):912-918.

8. Van der Auwera GA, Carneiro MO, Hartl C, et al. From FastQ data to high confidence variant calls: the Genome Analysis Toolkit best practices pipeline. *Curr Protoc Bioinformatics.* 2013;43:11 10 11-11 10 33.

9. McLaren W, Gil L, Hunt SE, et al. The Ensembl Variant Effect Predictor. *Genome Biol.* 2016;17(1):122.

10. O'Leary NA, Wright MW, Brister JR, et al. Reference sequence (RefSeq) database at NCBI: current status, taxonomic expansion, and functional annotation. *Nucleic Acids Res.* 2016;44(D1):D733-745.

11. Paila U, Chapman BA, Kirchner R, Quinlan AR. GEMINI: integrative exploration of genetic variation and genome annotations. *PLoS Comput Biol.* 2013;9(7):e1003153.

12. Richards S, Aziz N, Bale S, et al. Standards and guidelines for the interpretation of sequence variants: a joint consensus recommendation of the American College of Medical Genetics and Genomics and the Association for Molecular Pathology. *Genet Med.* 2015;17(5):405-424.

13. Stranneheim H, Engvall M, Naess K, et al. Rapid pulsed whole genome sequencing for comprehensive acute diagnostics of inborn errors of metabolism. *BMC Genomics.* 2014;15:1090.

14. Martin HC, Kim GE, Pagnamenta AT, et al. Clinical whole-genome sequencing in severe early-onset epilepsy reveals new genes and improves molecular diagnosis. *Hum Mol Genet.* 2014;23(12):3200-3211.

15. Alazami AM, Patel N, Shamseldin HE, et al. Accelerating novel candidate gene discovery in neurogenetic disorders via whole-exome sequencing of prescreened multiplex consanguineous families. *Cell Rep.* 2015;10(2):148-161.

16. Starr LJ, Spranger JW, Rao VK, Lutz R, Yetman AT. PIGQ glycosylphosphatidylinositol-anchored protein deficiency: Characterizing the phenotype. *Am J Med Genet A.* 2019;179(7):1270-1275.

**APPENDIX 1 Members of the Undiagnosed Diseases Network**

Maria T. Acosta

Margaret Adam

David R. Adams

Pankaj B. Agrawal

Mercedes E. Alejandro

Justin Alvey

Laura Amendola

Ashley Andrews

Euan A. Ashley

Mahshid S. Azamian

Carlos A. Bacino

Guney Bademci

Eva Baker

Ashok Balasubramanyam

Dustin Baldridge

Jim Bale

Michael Bamshad

Jordan Barham

Deborah Barbouth

Gabriel F. Batzli

Pinar Bayrak-Toydemir

Anita Beck

Alan H. Beggs

Gill Bejerano

Jimmy Bennet

Beverly Berg-Rood

Raphael Bernier

Jonathan A. Bernstein

Gerard T. Berry

Anna Bican

Stephanie Bivona

Elizabeth Blue

John Bohnsack

Carsten Bonnenmann

Devon Bonner

Lorenzo Botto

Brenna Boyd

Lauren C. Briere

Elly Brokamp

Gabrielle Brown

Elizabeth A. Burke

Lindsay C. Burrage

Manish J. Butte

Peter Byers

William E. Byrd

John Carey

Olveen Carrasquillo

Ta Chen Peter Chang

Sirisak Chanprasert

Hsiao-Tuan Chao

Mei-Jan Chen

Gary D. Clark

Terra R. Coakley

Laurel A. Cobban

Joy D. Cogan

F. Sessions Cole

Heather A. Colley

Cynthia M. Cooper

Heidi Cope

William J. Craigen

Michael Cunningham

Precilla D'Souza

Hongzheng Dai

Surendra Dasari

Mariska Davids

Jyoti G. Dayal

Esteban C. Dell'Angelica

Shweta U. Dhar

Katrina Dipple

Daniel Doherty

Naghmeh Dorrani

Emilie D. Douine

David D. Draper

Laura Duncan

Dawn Earl

David J. Eckstein

Lisa T. Emrick

Christine M. Eng

Cecilia Esteves

Tyra Estwick

Liliana Fernandez

Carlos Ferreira

Elizabeth L. Fieg

Paul G. Fisher

Brent L. Fogel

Irman Forghani

Laure Fresard

William A. Gahl

Ian Glass

Rena A. Godfrey

Katie Golden-Grant

Alica M. Goldman

David B. Goldstein

Alana Grajewski

Catherine A. Groden

Andrea L. Gropman

Sihoun Hahn

Rizwan Hamid

Neil A. Hanchard

Nichole Hayes

Frances High

Anne Hing

Fuki M. Hisama

Ingrid A. Holm

Jason Hom

Martha Horike-Pyne

Alden Huang

Yong Huang

Rosario Isasi

Fariha Jamal

Gail P. Jarvik

Jeffrey Jarvik

Suman Jayadev

Jean M. Johnston

Lefkothea Karaviti

Emily G. Kelley

Dana Kiley

Isaac S. Kohane

Jennefer N. Kohler

Deborah Krakow

Donna M. Krasnewich

Susan Korrick

Mary Koziura

Joel B. Krier

Seema R. Lalani

Byron Lam

Christina Lam

Brendan C. Lanpher

Ian R. Lanza

C. Christopher Lau

Kimberly LeBlanc

Brendan H. Lee

Hane Lee

Roy Levitt

Richard A. Lewis

Sharyn A. Lincoln

Pengfei Liu

Xue Zhong Liu

Nicola Longo

Sandra K. Loo

Joseph Loscalzo

Richard L. Maas

Ellen F. Macnamara

Calum A. MacRae

Valerie V. Maduro

Marta M. Majcherska

May Christine V. Malicdan

Laura A. Mamounas

Teri A. Manolio

Rong Mao

Kenneth Maravilla

Thomas C. Markello

Ronit Marom

Gabor Marth

Beth A. Martin

Martin G. Martin

Julian A. Martínez-Agosto

Shruti Marwaha

Jacob McCauley

Allyn McConkie-Rosell

Colleen E. McCormack

Alexa T. McCray

Elisabeth McGee

Heather Mefford

J. Lawrence Merritt

Matthew Might

Ghayda Mirzaa

Eva Morava-Kozicz

Paolo M. Moretti

Marie Morimoto

John J. Mulvihill

David R. Murdock

Mariko Nakano-Okuno

Avi Nath

Stan F. Nelson

John H. Newman

Sarah K. Nicholas

Deborah Nickerson

Donna Novacic

Devin Oglesbee

James P. Orengo

Laura Pace

Stephen Pak

J. Carl Pallais

Christina GS. Palmer

Jeanette C. Papp

Neil H. Parker

John A. Phillips III

Jennifer E. Posey

Lorraine Potocki

Barbara N. Pusey

Aaron Quinlan

Wendy Raskind

Archana N. Raja

Genecee Renteria

Chloe M. Reuter

Lynette Rives

Amy K. Robertson

Lance H. Rodan

Jill A. Rosenfeld

Natalie Rosenwasser

Robb K. Rowley

Maura Ruzhnikov

Ralph Sacco

Jacinda B. Sampson

Susan L. Samson

Mario Saporta

C. Ron Scott

Judy Schaechter

Timothy Schedl

Kelly Schoch

Daryl A. Scott

Lisa Shakachite

Prashant Sharma

Vandana Shashi

Jimann Shin

Rebecca Signer

Catherine H. Sillari

Edwin K. Silverman

Janet S. Sinsheimer

Kathy Sisco

Edward C. Smith

Kevin S. Smith

Lilianna Solnica-Krezel

Rebecca C. Spillmann

Joan M. Stoler

Nicholas Stong

Jennifer A. Sullivan

Angela Sun

Shirley Sutton

David A. Sweetser

Virginia Sybert

Holly K. Tabor

Cecelia P. Tamburro

Queenie K.-G. Tan

Mustafa Tekin

Fred Telischi

Willa Thorson

Cynthia J. Tifft

Camilo Toro

Alyssa A. Tran

Tiina K. Urv

Matt Velinder

Dave Viskochil

Tiphanie P. Vogel

Colleen E. Wahl

Stephanie Wallace

Nicole M. Walley

Chris A. Walsh

Melissa Walker

Jennifer Wambach

Jijun Wan

Lee-kai Wang

Michael F. Wangler

Patricia A. Ward

Daniel Wegner

Mark Wener

Tara Wenger

Katherine Wesseling Perry

Monte Westerfield

Matthew T. Wheeler

Anastasia L. Wise

Lynne A. Wolfe

Jeremy D. Woods

Shinya Yamamoto

John Yang

Guoyun Yu

Diane B. Zastrow

Chunli Zhao

Stephan Zuchner

1. Children’s Hospital of Eastern Ontario Research Institute, Ottawa, Ontario, Canada [↑](#footnote-ref-2)
2. Research Center, CHU Sainte Justine, University of Montreal, Montreal, Quebec, Canada [↑](#footnote-ref-3)
3. Department of Genetics, Children’s Hospital of Eastern Ontario, Ottawa, Ontario, Canada [↑](#footnote-ref-4)
4. Division of Metabolics and Newborn Screening, Department of Pediatrics, Children’s Hospital of Eastern Ontario, Ottawa, Ontario, Canada [↑](#footnote-ref-5)
5. Department of Human Genetics, McGill University, Montreal, Quebec, Canada [↑](#footnote-ref-6)
6. McGill University and Genome Quebec Innovation Centre, Montreal, Quebec, Canada [↑](#footnote-ref-7)
7. Division of Genetics, Department of Genetics, Kingston Health Sciences Centre Pediatrics, Queen’s University, Kingston, Ontario, Canada [↑](#footnote-ref-8)
8. Department of Neuropediatrics, Christian-Albrechts-University of Kiel, 24105 Kiel, Germany [↑](#footnote-ref-9)
9. Division of Human Genetics, Cincinnati Children’s Hospital Medical Center, Cincinnati, Ohio, 45229 [↑](#footnote-ref-10)
10. Department of Pediatrics, University of Cincinnati College of Medicine, Cincinnati, Ohio, USA [↑](#footnote-ref-11)
11. Department of Neurology, Academic Center for Epileptology Kempenhaeghe & Maastricht University Medical Center, Heeze, the Netherlands [↑](#footnote-ref-12)
12. Department of Pediatric Neurology, Amalia Children’s Hospital, Radboud University Medical Center & Donders Institute for Brain, Cognition and Behaviour, Radboud University, Nijmegen, the Netherlands [↑](#footnote-ref-13)
13. Division of Medical Genetics, A. I. DuPont Hospital for Children/ Nemours, Wilmington, Delaware, USA [↑](#footnote-ref-14)
14. Department of Pediatrics, Division of Human Genetics, Children’s Hospital of Philadelphia, Philadelphia, Pennsylvania, USA [↑](#footnote-ref-15)
15. Department of Pathology and Laboratory Medicine, Children’s Hospital of Philadelphia, Philadelphia, Pennsylvania, USA [↑](#footnote-ref-16)
16. Division of Medical Genetics, Department of Pediatrics, Duke University Medical Center, Durham, North Carolina, USA [↑](#footnote-ref-17)
17. Department of Pediatric Neurology, Region Skåne and Clinical Sciences, Lund University
    Skåne University Hospital (SUS), 221 85 Lund, Sweden [↑](#footnote-ref-18)
18. Department of Genetics and Personalized Medicine, Danish Epilepsy Centre, Dianalund, Denmark [↑](#footnote-ref-19)
19. Institute for Regional Health Services Research, University of Southern Denmark, Odense, Denmark [↑](#footnote-ref-20)
20. Division of Neurology, Department of Pediatrics, Children’s Hospital of Eastern Ontario, Ottawa, Ontario, Canada [↑](#footnote-ref-21)
21. Department of Pediatrics, Sainte-Justine Hospital, University of Montreal, Montreal, Quebec, Canada [↑](#footnote-ref-22)
